# The recent advancements in the early detection of cancer biomarkers by DNAzyme-assisted aptasensors

**DOI:** 10.1186/s12951-022-01640-1

**Published:** 2022-10-04

**Authors:** Hossein Kamali, Shiva Golmohammadzadeh, Hamed Zare, Rahim Nosrati, Mohammad Fereidouni, Hossein Safarpour

**Affiliations:** 1 Targeted Drug Delivery Research Center, Pharmaceutical Technology Institute, Mashhad University of Medical Sciences, Mashhad, Iran; 2 Nanotechnology Research Center, Pharmaceutical Technology Institute, Mashhad University of Medical Sciences, Mashhad, Iran; 3 Department of Pharmaceutics, School of Pharmacy, Mashhad University of Medical Sciences, Mashhad, Iran; 4 Recombinant Proteins Department, Breast Cancer Research Center, Motamed Cancer Institute, ACECR, Tehran, Iran; 5 Cellular and Molecular Research Center, School of Medicine, Guilan University of Medical Sciences, Rasht, Iran; 6 Cellular and Molecular Research Center, Birjand University of Medical Sciences, Birjand, Iran

**Keywords:** DNAzyme, Aptasensor, Biosensor, Nanoparticle, Cancer biomarker

## Abstract

**Abstract:**

Clinical diagnostics rely heavily on the detection and quantification of cancer biomarkers. The rapid detection of cancer-specific biomarkers is of great importance in the early diagnosis of cancers and plays a crucial role in the subsequent treatments. There are several different detection techniques available today for detecting cancer biomarkers. Because of target-related conformational alterations, high stability, and target variety, aptamers have received considerable interest as a biosensing system component. To date, several sensitivity-enhancement strategies have been used with a broad spectrum of nanomaterials and nanoparticles (NPs) to improve the limit and sensitivity of analyte detection in the construction of innovative aptasensors. The present article aims to outline the research developments on the potential of DNAzymes-based aptasensors for cancer biomarker detection.

**Graphical Abstract:**

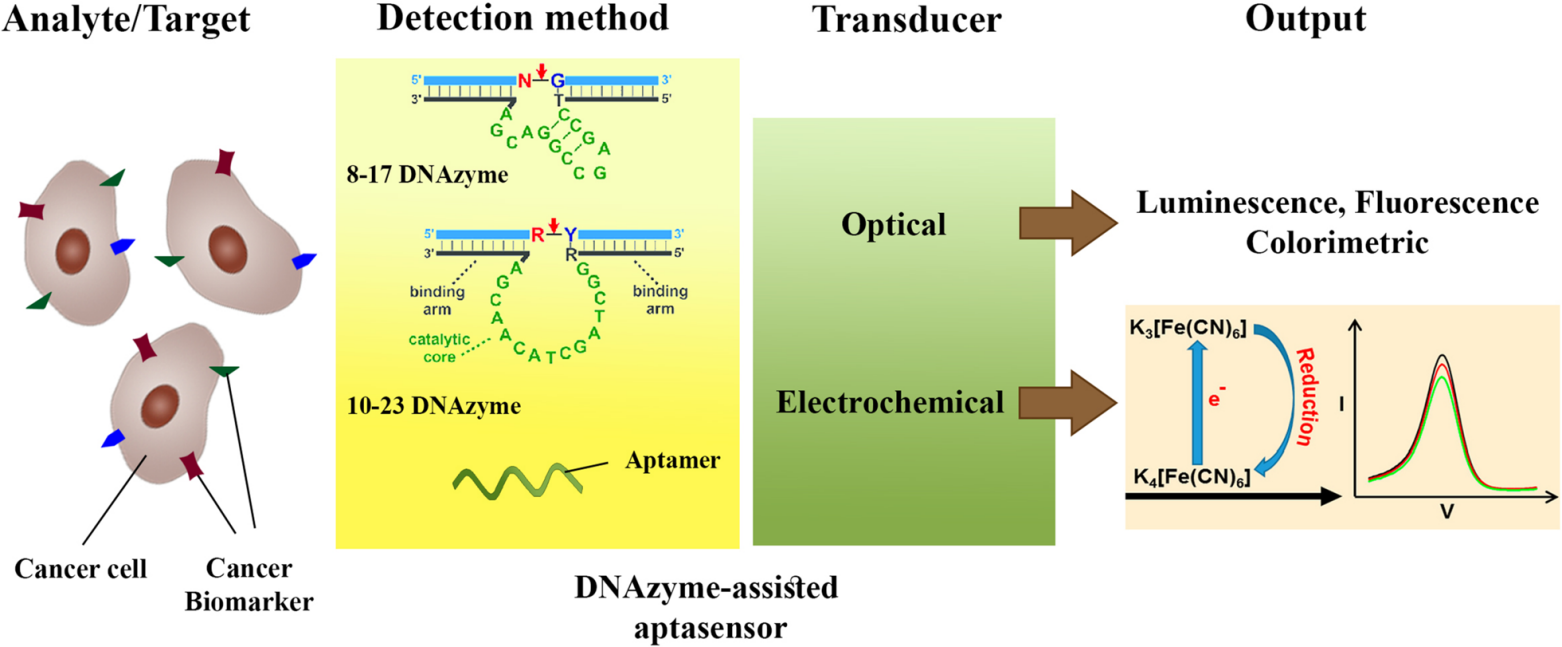

## Introduction

Cancer is one of the main causes of mortality in industrialized and developing countries [1]. Early diagnosis significantly reduces cancer-related mortality [2]. Currently, morphological investigation of tissues (histopathology) or cells (cytology), and imaging tools help with early cancer detection. The majority of imaging methods, including X-rays, magnetic resonance imaging (MRI), computed tomography (CT), endoscopies, and ultrasounds, can only identify cancer after the tissue has undergone a visible alteration [1]. At that point, cancer cells may have already multiplied and even spread to other parts of the body, a process named metastasis. In addition, the available imaging techniques can not differentiate between benign and malignant tumors [3]. Furthermore, cytology and histopathology cannot be used successfully and independently to diagnose cancer in its early stages [4]. As a result, the development of new technologies that can identify cancer at an early stage, before it has spread to other parts of the body, presents a significant challenge.

A cancer biomarker is a biological molecule that can be measured and is indicative of the presence of cancer in the body. Cancer biomarkers may be discovered in blood as well as other tissues or body fluids like saliva and urine [5, 6]. It is possible to identify cancer or tumor recurrence early thanks to the measurement of specific cancer biomarker levels, which also aids in assessing how well the treatment is working [7, 8]. In this respect, protein biomarkers are appealing candidates for detection since they directly reflect the phenotype and the pathophysiological mechanisms of cancer cells [9, 10]. However, some obstacles have restricted the use of biomarkers, including low biomarker concentrations in body fluids, variability in the quantity and timing of biomarkers among individuals, and the difficulty of conducting prospective research [11].

Nanotechnology enables very high selectivity and sensitivity, as well as the capacity to perform simultaneous tests on different targets. The ability of biosensors to enable precise targeting may be increased by including nanomaterials (material with at least one internal or external structure on the nanoscale dimension) and nanoparticles (NPs) (nano-object with three external nanoscale dimensions) [12, 13]. NPs boost a high surface-to-volume ratio, making biosensors more sensitive to biomolecular diagnostics [14]. Because of this feature, antibodies, small molecules, peptides, aptamers, DNAzymes, and other moieties may be extensively coated on NPs surfaces [15]. These compounds can attach to and identify certain cancer molecules. Multivalent effects may be obtained by introducing multiple binding ligands to cancer cells, which can increase the specificity and sensitivity of a test [1].

### DNAzyme

DNAzymes (also known as catalytic DNA) have been shown to selectively execute activities such as target mRNA degradation, ligation, or phosphorylation of DNA [16]. Many different forms of DNAzymes have been discovered using in vitro screening technologies and have a great structural recognition capacity [17]. Among them, three groups of DNAzyme, including RNA-cleaving DNAzymes, DNA-cleaving DNAzymes, and Hemin/G-quadruplex (G4) DNAzyme, have significant potential in cancer biomarker detection approaches [18, 19]. DNAzyme contains a central catalytic zone that is surrounded by two-sided recognition arms. The longer recognizing more robust arm binds to its substrate *via* DNAzyme’s Watson-Crick base-pairing [20]. In this approach, the unique sequence of recognition arms defines the specificity of the DNAzyme and stabilizes the DNAzyme’s catalytic core, allowing the hydrolysis event to take place [17, 19]. The most common forms of DNAzyme are 8–17 and 10–23 DNAzyme, which may break practically any phosphodiester link between unpaired purines and paired pyrimidines [20, 21]. DNAzyme may successfully cut off the target mRNA in the presence of particular metal ions including Mg^2+^, Pb^2+^, Mn^2+^, Cu^2+^, and Na^+^ by catalyzing the hydrolysis of the target RNA’s phosphodiester [20, 22]. Therefore, DNAzyme might become a promising revolutionary gene therapy strategy and a powerful tool for the fabrication of biosensing platforms for cancer diagnosis.

### Aptamer and aptasensors

Aptamers are RNA or DNA strands synthesized by SELEX (Systematic evolution of ligands by exponential enrichment) that may be specifically linked to cells, proteins, and molecules [23]. Aptamers have numerous benefits over antibodies, including increased stability and resistance to environmental conditions, simpler production, and easier storage [24, 25]. Aptamers, because of their remarkable flexibility, can be bound to their target in a wholly precise manner. As a result, they are extensively used in biosensing technologies for identifying biological components, different diseases, and biomarkers [26, 27].

Aptamer-based biosensors (aptasensors) are devices that indicate the existence or quantity of a chemical or biological ingredient and offer both quantitative and qualitative data [28]. An aptasensor includes an aptamer as a bioreceptor that detects a target and generates a signal, as well as a signal transduction element, which is often optical or electrochemical, and a display system. Aptasensors have gained increasing interest in the last twenty years owing to their low cost, great sensitivity, reliability, and speed of recognition [29, 30]. Furthermore, the insertion of NPs into aptasensors results in nano-aptasensors, which have opened up new, more accurate detection methods in some different disciplines with great sensitivity [31]. From this perspective, the nanomaterials-based DNAzyme (also known as aptazyme) provides advanced biosensors for prognostics, diagnostics, and therapeutic screening of diseases [32]. The potential of DNAzyme-based aptasensors for the sensing of cancer-specific biomarkers is discussed in the present article. Furthermore, breakthroughs in the manufacturing of NPs-based aptazymes for cancer biomarker detection are highlighted.

## DNAzymes-assisted aptasensors for cancer detection

DNAzyme-assisted aptasensors are synthesized entities consisting of an aptamer structure and a catalytically active nucleic acid unit, which can be a ribozyme or a DNAzyme [21, 33]. The aptamer domain acts as a molecular switch in these structures, regulating the catalytic activity of the DNAzyme component [34]. The aptamer-target binding induces major physical transformations in the aptamer structure and hence in the complete aptazyme. As a result, aptazymes behave similarly to allosteric enzymes, in which catalytic activity is controlled by the binding of ligands (effectors) to allosteric sites caused by alterations in the 3D structure of the enzyme’s active site [33, 34]. The allosteric site of aptazymes is made up of an aptamer. Aptazymes may be created for a variety of purposes and have previously been employed in analytical tests as well as gene expression regulation [21].

### Optical-based DNAzymes-assisted aptasensors

#### Luminescence-based DNAzymes-assisted aptasensors

Currently, several optical-based DNAzyme-assisted aptasensors are reported for the detection of cancer. Luminescence-based platforms are one of them. The phenomenon of luminescence occurs when a material emits light that is not caused by heat. In a vast variety of light emission procedures, the luminescence-based techniques are categorized in accordance with the source of energy that triggers the luminescence [35]. Chemical processes are the source of energy for chemiluminescence (CL). Electrochemiluminescence (ECL) is also created in solutions during electrochemical processes [36]. Luminescence-based methods are considered a widespread platform for the construction of ultrasensitive biosensors due to significant benefits, for instance, high sensitivity, superior selectivity, and a broad linear range (LR). Some researchers have been interested in using luminescence-based DNAzyme aptasensing platforms for biomarker-based cancer detection (Table 1). Most of them were targeted at carcinoembryonic antigen (CEA) and platelet-derived growth factor (PDGF).

**Table 1:** Performance of luminescence and fluorescence-based DNAzyme-assisted aptasensors for cancer detection

Detection Method	Biomarkers/cells	Strategy	Sample	Aptamer Sequence (5′ → 3′)	NM/NPs	LOD	LR	Refs.
Luminescence	CEA	A quenching probe containing DNAzyme/ AuNRs-cDNA	Serum	NH_2_-(CH_2_)_6_-TTTTATACCAGCT TATTCAATT	AuNRs	0.036 pg mL^− 1^	0.1 pg mL^− 1^ − 0.5 ng mL^− 1^	[41]
		A dual amplification by CHA and HCR	Serum	TACCAGCTTATTCAATT	-	8 fg mL^− 1^	8 fg mL^− 1^ − 0.50 pg mL^− 1^	[42]
		a dual CEA/hemin conjugated aptamers/HRP-like G4-DNAzyme	Serum/Blood	CH-1: AAAGGTAGGGCGGGT TGGGTAAATAAAAAAGGGGGT GAAGGGATACCC CH-2: TACCAGCTTATTCAATTA AAAATAAAGGGTAGG GCGGGTTGGGTAAAT	-	0.58 ng mL^− 1^	0-200 ng mL^− 1^	[43]
		Pb^2+^-assisted DNAzyme/GQDs-IL-NF nanocomposite	Plasma	CATCTCTTCTCCGAGCCGGTC GAAATAGTGAGTATACCAGCTTA TTCAATTAAGAGATG	GQDs	0.34 fg mL^− 1^	0.5 fg mL^− 1^ to 0.5 ng mL^− 1^	[44]
		graphitic carbon nitride (g-C_3_N_4_) nanosheets and DNAzyme	Serum	GAATAAGCTTCCACCA TCCATACCAGCTTATTCTATT	g-C_3_N_4_ nanosheets	63.0 pg mL^− 1^	0.1–150 ng mL^− 1^	[49]
	PDGF	Exo III-CRA based label-free CL aptasensors	Serum	CTCAGGCTACGGCACGTAGAG CATCACCATGATCCTGAG	-	6.8 × 10^− 13^ M	-	[55]
	VEGF	T7 exonuclease, CdS:Eu NCs, andG4/hemin DNAzyme	Blood	GGCCCGTCCGTATGGTGG GTGTGCTGGCC	CdS:Eu nanocrystals	0.2 pM	1 pM to 20 nM	[62]
	PSA	MOF/Au/G4 DNAzyme	Serum	(CH_2_)6-TTTTTAATTAAA GCTCGCCATCAAATAGCTTT	AuNPs	0.058 ng mL^− 1^	0.5 to 500 ng mL^− 1^	[64]
	miR-205	G4 DNAzyme/ ODI-CL/Amplex Red/H_2_O_2_	Serum/Blood	UCCUUCAUUCCA CCGGAGUCUG	-	0.13 nM	0.4–62.5 nM	[70]
	miR-155 miR-21 p53/BRCA1 genes	CRET-based method/ UiO-66 MOF-NPs/luminol/H_2_O_2_	Serum	miR-155: UUAAUGCUAAUCGU GAUAGGGGU miR-21: UAGCUUAUCAGAC UGAUGUUGA p53: TCATCACACTGGAAGACTC BRCA1:AAAGTGTTTTTCATAAACCCATTATCCAGGACTGTTTATAGCTGTTGGAAG	MOF-NPs	-	-	[71]
	BRCA1	HRP-mimic DNAzyme	Buffer	AGGGCGGGTGGGTGTTTAAGTT GGAGAATTGTACTT AAACACCTTCTTCTTGGGT	-	1 × 10^− 13^ M	-	[75]
	Exosomal biomarker	MPA-CdS:Eu NCs/DNAzyme	Plasma	CD63 aptamer: CACC CCACCTCGCTCCCGTGA CACTAA TGCTATTTTTTTTTT-(CH_2_)_7_-NH_2_	CdS:Eu nanocrystals	7.41 × 10^4^ particle mL^− 1^	3.4 × 10^5^ to 1.7 × 10^8^ particle mL^− 1^	[78]
Fluorescence	BRCA1	Mg^2+^-dependent DNAzyme	Buffer	TTGCTCCCTGTTGCTGAAAC CATACAGCTTCATAAATA ATTTTGCTT	-	1 × 10^− 14^ M	-	[81]
	PDGF	PET between DNA-Ag NCs and G4/hemin	Serum	CAGGCTACGGCACGTAGAGCATCACCATGATCCTGTTTT	Ag NCs	1 × 10^13^ M	5 × 10^13^ to 1 × 10^8^ M	[82]

##### Carcinoembryonic antigen (CEA)

CEA is a kind of protein that can typically only be found in adult blood in very trace concentrations [37]. An increase in CEA blood levels may be brought on by some types of cancer. CEA has been recommended as a potential prognostic marker in a number of malignancies, both for the diagnosis of tumors and the monitoring of therapeutic response [38]. It has been shown that high CEA levels are particularly associated with the progression of cancer, and it has been expected that increased levels of the marker will fall after surgery [39]. Because of its role in the development of cancer, CEA has been suggested as a possible therapeutic and diagnostic target for cancer [39, 40].

**Fig. 1:**
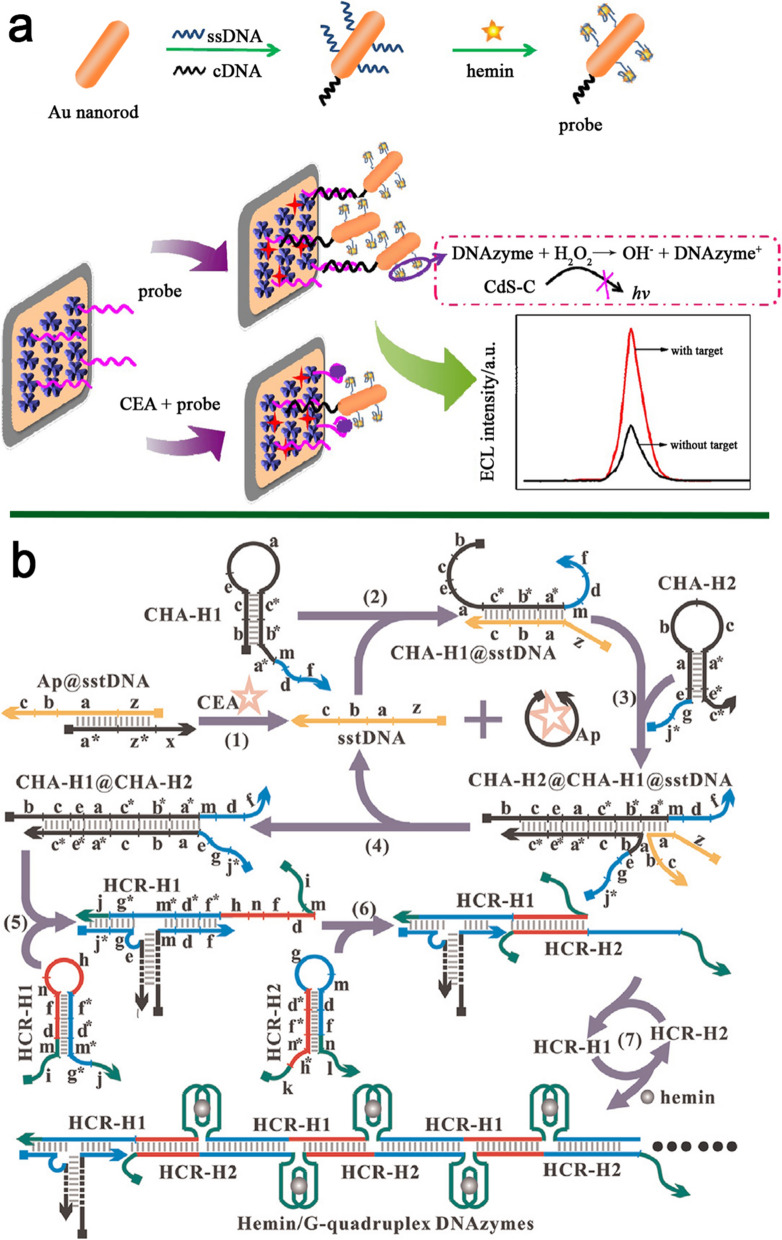
**a** Graphic depiction of an ECL aptasensor based on DNAzyme AUNRs for CEA detection; **b** Graphical illustration of a PEC-based aptazyme for the identification of CEA through CHA and HCR amplification. Reproduced with permission from [41] and [42]

Cao and colleagues designed an ECL-assisted aptasensor for CEA detection using a quenching probe containing DNAzyme/gold nanorods (AuNRs)-complementary DNA (cDNA). Then, the quenching probe was reacted with hemin to form hemin/G4 DNAzyme units. The ECL emitter (carbon-coated petalous CdS NPs (CdS-C petalous NPs)) was used as a matrix for the manufacture of the CdS-C/Chit/aptamer system. Without CEA, DNAzyme/AuNRs can be immobilized on the aptasensor surface by hybridization with an aptamer. In this condition, the DNAzyme catalyzed the reduction of H_2_O_2_, resulting in lower ECL emission. In the presence of CEA and quenching probe, their competitive reaction with the capture aptamer located on the electrode decreased the quantity of quenching probe, which resulted in a reduction in the H_2_O_2_ consumption, causing the amplified ECL signal (Fig. 1a). Their aptasensor detected CEA with a sensitivity of an LR of 0.1 pg mL^− 1^ − 0.5 ng mL^− 1^ with a limit of detection (LOD) of 0.036 pg mL^− 1^. The LOD in human sera was 0.21 pg mL^− 1^, with recoveries ranging from 88.2 to 106% [41]. In another research, for the first time, an ultrasensitive photo ECL (PEC) aptasensor was constructed using a unique enzyme-free cascaded quadratic signal amplification approach (Fig. 1b). This dual amplification method comprises a target-analog recycling circuit based on catalytic hairpin assembly (CHA) and a hybridization chain reaction (HCR)-mediated amplification. The release of target-analog in the existence of CEA activated the upstream CHA and downstream HCR amplification, resulting in the creation of hemin/G4 DNAzymes. As a result, the biocatalytic precipitation of 4-chloro-1-naphthol caused a reduction in the photocurrent signal. This PEC DNAzyme-assisted aptasensor detected the atto-gram (10^− 18^ gram) level of CEA [42].

**Fig. 2:**
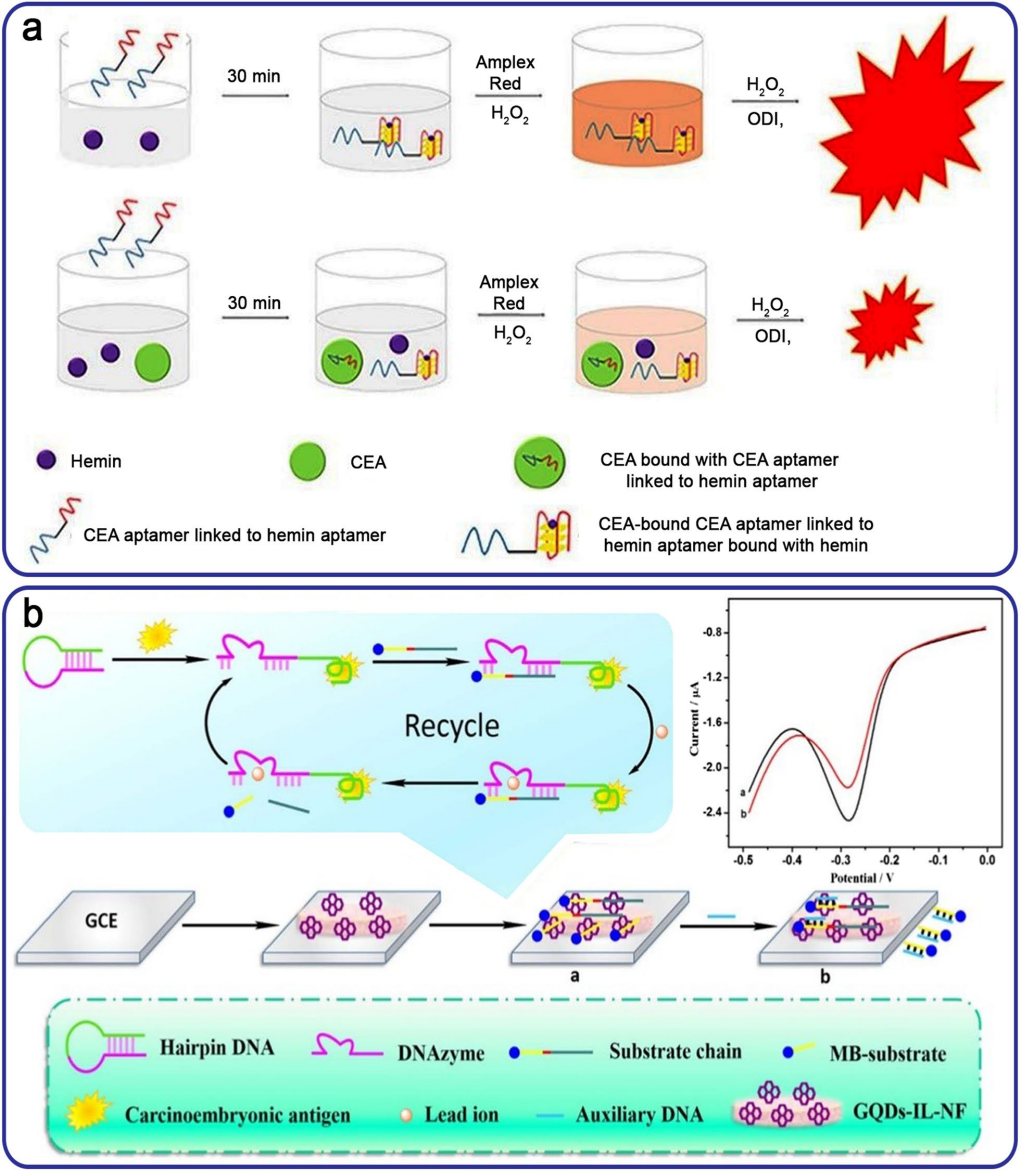
**a** A detection technique for CEA identification based on a DNAzyme dual-aptasensor; **b** An ECL-based detection of CEA using the GQDs-IL-NF nanocomposite and DNAzyme. Reproduced with permission from [43] and [44]

A DNAzyme-aptasensor was created using dual CEA/hemin conjugated aptamers for the CL-based detection of CEA with 1,1′-oxalyldiimidazole (ODI). In this method, the interaction of Amplex Red and H_2_O_2_ produced the resorufin, which was dependent on the quantity of HRP-like G4-DNAzyme. After the addition of ODI and H_2_O_2_ to the resorufin-containing target cell, a bright red light was detected (Fig. 2a). In a blood sample containing CEA, the linking of dual-aptamer to CEA decreased the relative CL intensity of the dual-aptasensor. The aptasensor’s LOD was 0.58 ng mL^− 1^, demonstrating high accuracy, precision, and repeatability [43]. In 2018, a research team proposed an ECL-based aptasensor for the quick identification of CEA *via* lead ion (Pb^2+^)-assisted DNAzyme signal amplification and graphene quantum dot (GQDs)-ionic liquid-nafion (GQDs-IL-NF) composite film. They created hairpin DNA using CEA-binding aptamers and DNAzyme sequences. The identification of CEA by hairpin DNA executed a DNAzyme-based signal amplification process and the release of ssDNA. Then, the deposited GQDs-IL-NF on a glassy carbon electrode (GCE) interacted with ssDNA. As a result, the methylene blue (MB)-labeled substrate DNA was attached to the electrode and produced an ECL signal (Fig. 2b). The reaction current change was related to the CEA level, with a wide LR of 0.5 fg mL^− 1^ to 0.5 ng mL^− 1^ and a LOD of 0.34 fg mL^− 1^ [44]. QDs have been mostly utilized as luminescence materials due to specific characteristics, including size-dependent emission [45, 46]. It is important to note the toxicity and chemical instability of QDs in aptasensing platforms [35].

**Fig. 3:**
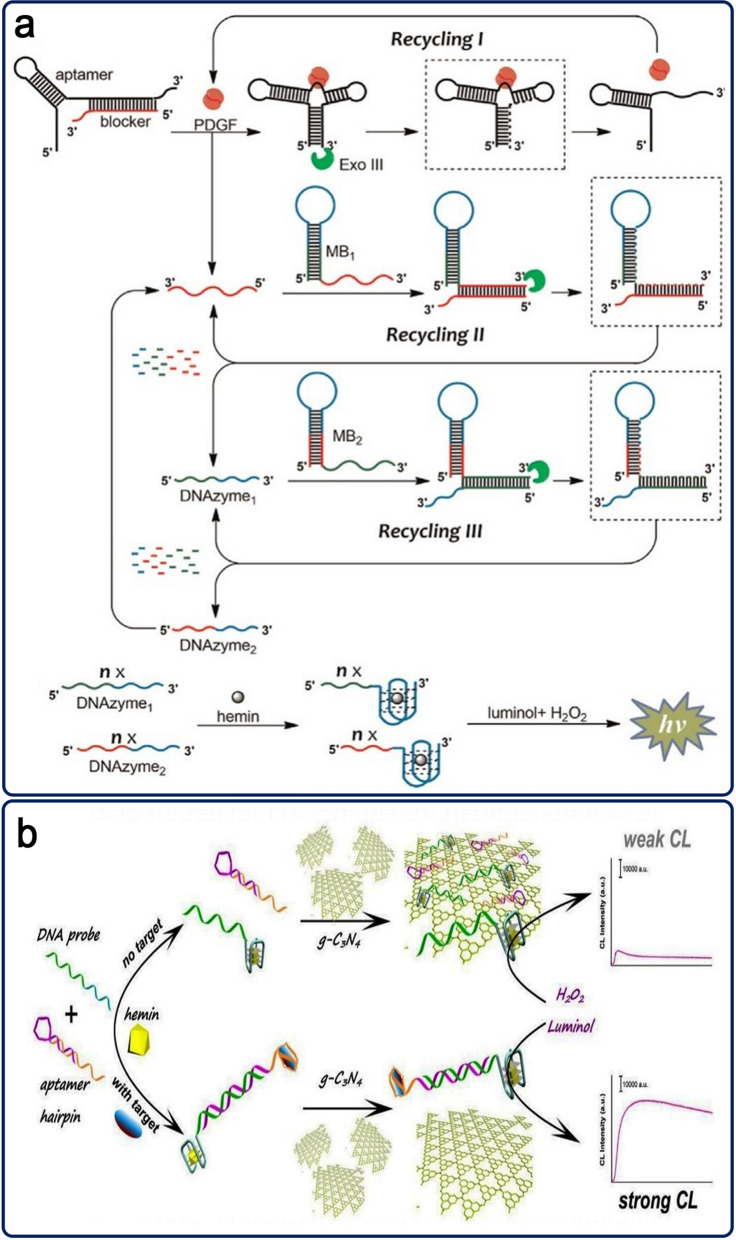
**a** The CEA detection process based on g-C3N4 NS and luminol-hemin/G4 DNAzyme CL aptasensors; **b** An Exo III-CRA based label-free CL aptasensors for PDGF-BB detection. Reproduced with permission from [49] and [55]

Because of their peculiar electrical structure and exceptional characteristics, graphitic carbon nitride (g-C_3_N_4_) nanosheets (NS), which are typical metal-free semiconductors with a layered structure, have found use in the fields of the environment, energy, and biology [47]. The g-C3N4 NS, coated with several functional groups, was used as a versatile material in photocatalytic applications as well as a photo-induced indicator [48]. Li and coworkers presented a label-free and homogenous CL aptasensing scaffold using g-C3N4 NS and DNAzyme for sensitive CEA detection (Fig. 3a). In the absence of CEA, a DNA probe with a hemin/G4 DNAzyme structure was immobilized on the g-C3N4 NS surface, enabling the quenching of CL signal. In contrast, the CEA was hybridized with a DNA-DNA duplex and was not able to adsorb on the g-C3N4 NS surface due to its low affinity. As a result, electron transfer was inhibited, allowing luminol’s CL emission to be boosted. The suggested CL aptasensor has a LOD of 63.0 pg mL^− 1^ for CEA [49].

##### Platelet-derived growth factor (PDGF)

PDGF refers to a family of growth factors that control cell development and division. PDGF is a dimeric glycoprotein with two similar A subunits (PDGF-AA), two B chains (PDGF-BB), and an AB heterodimer including PDGF-AB and PDGF-BA [50]. Although PDGF BB is more powerful than PDGF AA, and PDGF AB has intermediate activity, they have similar biological activities. It has long been recognized that PDGF-BB promotes ligament fibroblast proliferation and migration. In epithelial malignancies, paracrine PDGF signaling drives stromal recruiting and could be implicated in epithelial-mesenchymal transition (EMT), impacting tumor development [50, 51]. The proliferation of cancer, metastasis, invasion, and angiogenesis are all associated with the activation of the PDGF/PDGFR signaling pathway [52, 53]. A high level of PDGF expression has been linked to several cancers as well as non-cancer conditions defined by excessive cell proliferation, such as fibrotic disorders, atherosclerosis, and dysplasia [54].

Bi et al. (2014) proposed a CL-based on exonuclease III (ExoIII)-mediated cascade recycling amplification (Exo-CRA) method for detecting PDGF-BB that makes use of the aptamer unit and Exo III cleavage function. This system is made up of a duplex DNA (aptamer–blocker hybrid), MB1 and MB2 as hairpin structures, and ExoIII (Fig. 3b). The aptamer-PDGF-BB binding triggered the hypothesized Exo-CRA response. Lastly, hemin intercalated into G4/DNAzymes conjugated to catalyze luminol oxidation by H_2_O_2_ and produced an enhanced CL signal with a LOD 6.8 × 10^− 13^ M PDGF-BB. The simplicity, isothermal situations, homogeneous reaction lacking separation and washing phases, low cost, and increased amplification performance make this an aptasensor as a suitable detection method [55].

##### Vascular endothelial growth factor (VEGF)

VEGF is a critical regulator of normal and abnormal embryonic vasculogenesis and adult angiogenesis [56]. The prominent isoform, VEGF_165_, is responsible for the development and spread of a number of malignant malignancies [57, 58]. It is involved in pathological angiogenesis, which is linked to a variety of cancers and other diseases. As a result, a high level of VEGF might be a sign of cancer [59, 60]. The mRNA and serum VEGF levels have been shown to rise in tandem with the rate of metastasis in a number of studies [61]. This is likely since VEGF plays a role in the formation of new blood vessels, which in turn supply cancer cells with the nutrients and oxygen they need to thrive and spread. Given the importance of VEGF’s functions, this biomolecule has drawn a lot of attention for sensing and identifying it as a biomarker using analytical sensing systems. Recently, some DNAzyme aptasensors for VEGF detection have been demonstrated.

**Fig. 4:**
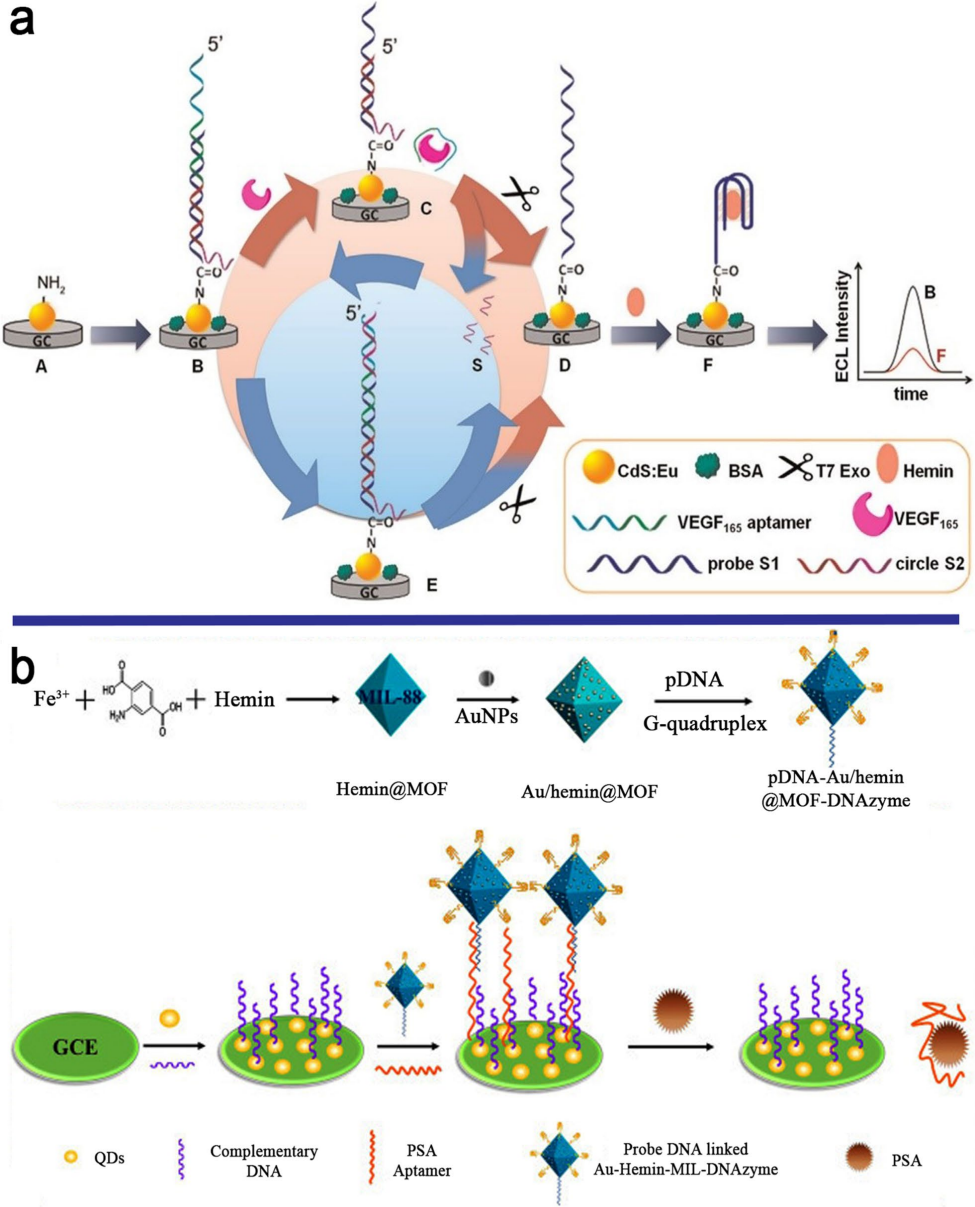
**a** A VEGF_165_ ECL detection method based on T7 exonuclease, CdS:Eu NCs, andG4/hemin DNAzyme; **b** The detection of PSA by ECL aptasensor including MOF/Au/G4 DNAzyme. Reproduced with permission from [62] and [64]

In a work, an ECL method for precise identification of VEGF_165_ was reported based on T7 exonuclease (T7 Exo)-mediated cycling signal amplification and DNAzyme-assisted ECL quenching of CdS:Eu nanocrystals (NCs) (Fig. 4a). This strategy benefits from the VEGF_165_ aptamer and a guanine (G)-rich single-stranded DNA (ssDNA) sequence that were simultaneously decorated on the surface of the CdS:Eu NCs-coated GCE. The binding of VEGF_165_ to its aptamer deposited the aptamer on the GCE surface and triggered the cutting of the target DNA with T7 Exo. In the presence of hemin and K^+^, a huge level of G-rich ssDNA was exposed to the CdS:Eu film and enveloped into G4/hemin DNAzyme, lowering the ECL intensity of CdS:Eu. The detection of VEGF_165_ was found to have a good LR of 1 pM to 20 nM with a LOD of 0.2 pM [62].

##### Prostatic specific antigen (PSA)

PSA is a substance secreted by the prostate gland. High PSA levels can be a good marker for prostatic diseases, including prostate cancer, benign prostatic hyperplasia, prostatitis, or an enlarged prostate gland. This protein is a blood-based cancer biomarker that is utilized in all stages of prostate cancer detection and patient treatment, including screening, risk assessment for recurrence, surveillance after diagnosis, and therapy monitoring [63]. Shao et al. developed a new MOF/Au/G4 as both quenchers and enhancers to create a target-triggered signal-amplified ratiometric ECL (Sa-RECL) assay platform capable of detecting PSA with high sensitivity and accuracy (Fig. 4b). Sa-RECL is an appealing strategy for increasing the signal-to-noise ratio by amplifying signals and eliminating interference. In this ratiometric ECL, dual ECL emitters (QDs and luminol) and a MOF/Au/G4 play as amplification probes. Following consecutive hybridization of complementary DNA-QDs, PSA aptamer, and pDNA-Au-Hemin-MIL-DNAzyme, and subsequent PSA competition, the pDNA-Au-Hemin-MIL-DNAzyme probe would remain away from the electrode surface, reducing the ECL signals. The ratiometric ECL aptasensor detected PSA with an LR of 0.5 to 500 ng mL^− 1^ and a LOD of 0.058 ng mL^− 1^ [64].

##### Cancer-related MicroRNAs and genes

MicroRNAs (miRNAs) have been discussed in the literature as possible biomarkers for a variety of cancers [65]. miRNAs are non-coding RNAs (ncRNAs) with 20–24 nucleotides in length that regulate gene expression in multicellular organisms by altering the stability and translation of mRNAs [66, 67]. The miR-205 is functioned as a tumor suppressor and is promoted tumor metastasis due to its role in the epithelial to mesenchymal transition [68, 69]. Kim et al. used a G4 DNAzyme connected to a complementary probe and ODI chemiluminescence (ODI-CL) to quantify trace amounts of microRNA-205 as a lung cancer biomarker. In this platform, the captured complementary probes on the surface of a paramagnetic bead are specifically attached to microRNA-205 in human serum. The produced complex connected to the hemin aptamer, and its interaction with hemin led to the fabrication of a G4/hemin DNAzyme. This was used as an HRP-mimicking DNAzyme that produced resorufin *via* the interaction of Amplex Red with H_2_O_2_. The amount of produced resorufin was proportional to the amount of microRNA-205 in human blood. Thus, increasing microRNA-205 elevated the brightness of released resorufin in the ODI-CL reaction. The aptasensor is capable of sensing as low as 0.13 nM microRNA-205 with an LR of 0.4–62.5 nM [70].

A CL resonance energy transfer (CRET) based on functional hybrid modules consists of dye-loaded UiO-66 MOF-NPs equipped with catalytic hemin/G4 DNAzyme tags for the assessment of miRNA-155 or miRNA-21 and the p53 or BRCA1 genes. In the presence of the targets, the hemin/G4 DNAzyme connected to the dye-loaded MOF-NPs. The existence of luminol and H_2_O_2_, DNAzyme generated by CL that delivered radiative energy to stimulate the CRET to the dye loaded in the MOF-NPs, led to the luminescence of the loaded dye with no additional excitation. The produced CRET signals are related to the concentrations of the genes or the miRNAs [71].

BRCA1 is a human tumor suppressor gene (sometimes referred to as a caretaker gene) that is in charge of DNA repair. BRCA1 and BRCA2 are unrelated proteins that are routinely produced in breast and other tissue cells, where they help repair damaged DNA or kill cells if DNA repair is not possible [72, 73]. BRCA1/2 has received a lot of interest recently as a biomarker for predicting cancer prognosis. The discovery of biomarkers such as BRCA1/2 is critical in advancing the precise therapy of triple-negative breast cancer (TNBC) [74]. In a similar study, an HRP-mimic DNAzyme CL amplified platform was provided to detect BRCA1. In the existence of the BRCA1 DNA, the opening of hairpin 1 triggered the opening of hairpin 2 *via* the strand displacement principle, which led to the construction of nanowires containing the HRP-like DNAzyme. The DNA nanowires acted as catalytic tags for the colorimetric or CL readout of the sensing procedures. This platform detected the BRCA1 with a LOD of 1 × 10^− 13^ M [75].

##### Exosomal biomarkers

**Fig. 5:**
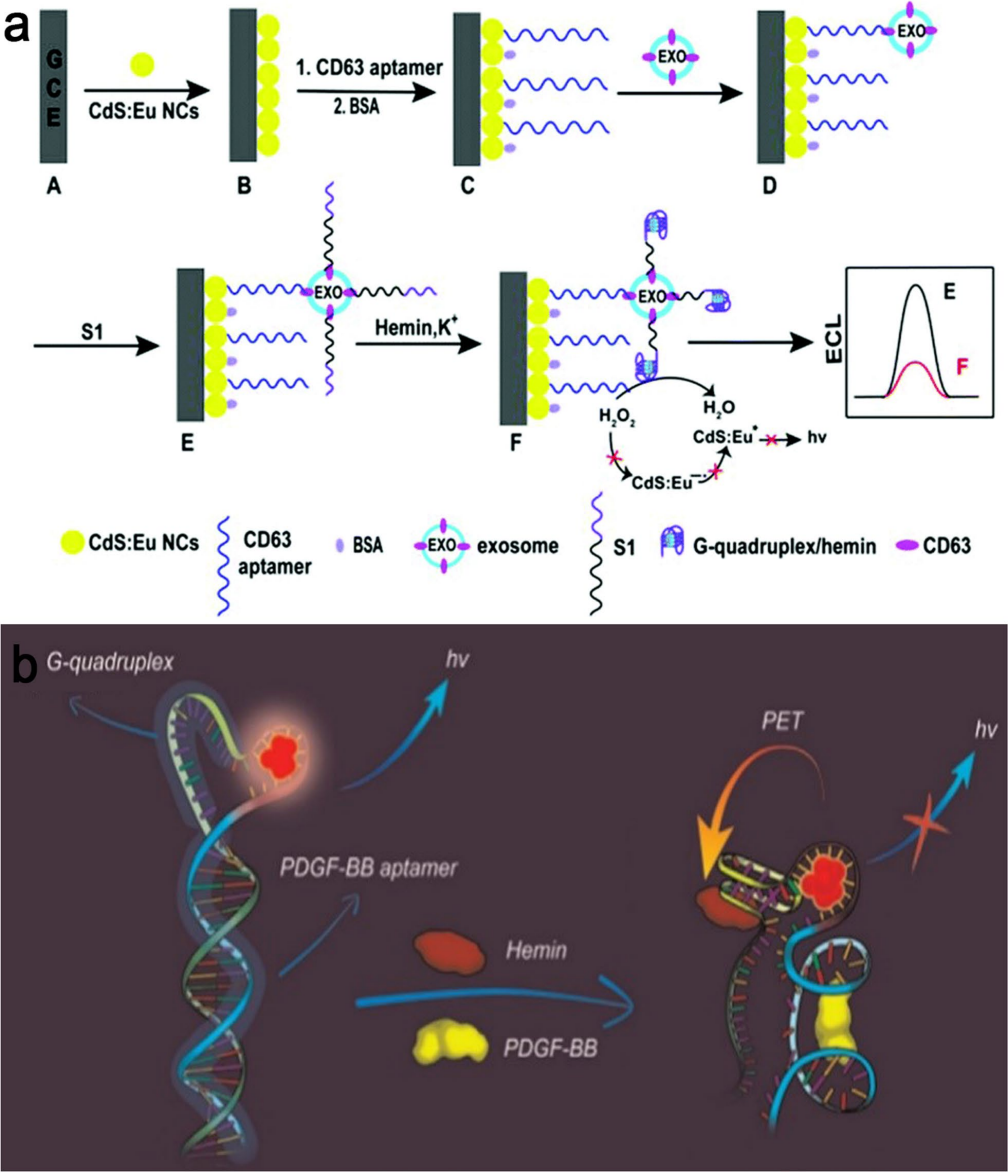
**a** An ECL-based aptasensing of exosome detection MPA-CdS:Eu NCs and DNAzyme; **b** A fluorescence-based DNAzyme containing AG NCs for PDGF-BB detection. Reproduced with permission from [78] and [82]

Recently, extensive research has been conducted on using exosomes as biomarkers for cancer detection. Studies have also found that exosomes may be vital sources of cancer biomarkers. Therefore, exosomal biomarkers have the potential to be innovative targets in cancer detection and prognosis [76]. Exosome components, such as proteins, DNA, coding and noncoding RNAs, circular RNA, and so on, play an important role in regulating tumor growth, metastasis, and angiogenesis during the cancer development process [77]. An exosome-detecting ECL aptasensor from breast carcinoma cells was designed. ECL emitters and co-reactants were mercaptopropionic acid (MPA)-modified Eu^3+^-doped CdS nanocrystals (MPA-CdS:Eu NCs) and H_2_O_2_, respectively. The CD63 aptamer recognized and captured exosomes and subsequently produces G4/hemin DNAzyme, which effectively catalyzed the breakdown of H_2_O_2_, leading to a diminished ECL signal of MPA-CdS:Eu NCs (Fig. 5a). MCF-7 cell-released exosomes can be identified at LR from 3.4 × 10^5^ to 1.7 × 10^8^ particles mL^− 1^ with a LOD of 7.41 × 10^4^ particles mL^− 1^ [78].

#### Fluorescence-based DNAzymes-assisted aptasensors

Fluorescence is a subcategory of luminescence. The detection methods based on fluorescence are among the most important optical strategies for the construction of diverse aptasensors owing to their high sensitivity, repeatability, quick procedure, and non-destructiveness [79, 80]. Several fascinating investigations are underway in this area, intending to use fluorescence-based technologies to identify cancer biomarkers.

In a study, BRCA1 was optically detected by Mg^2+^-dependent DNAzyme and the respective fluorophore/quencher-modified substrate with a sensitivity corresponding to 1 × 10^− 14^ M [81]. Wang et al. developed a fluorescence-based label-free aptasensor for PDGF-BB *via* the photoinduced electron transfer (PET) between DNA-Ag fluorescent nanoclusters (NCs) and G4/hemin complexes (Fig. 5b). When adding the PDGF-BB, selective interaction with its aptamer caused the duplex-like DNA sequence to shift conformation, allowing the release of the G4 sequence portion. The existence of hemin and K^+^ produced the HRP-mimicking DNAzyme (HRP-DNAzyme). The PET happened with a reduction in the fluorescence intensity of the DNA-Ag NCs due to the transfer of electrons between the DNA-Ag NCs and the hemin core of the HRP-DNAzyme. In fact, the quenching mode was induced by PDGF-BB and hemin. The proposed probe was nontoxic and label-free, requiring only one-step hybridization. The findings revealed PDGF-BB selectivity and sensitivity with an LR of 5 × 10^13^ to 1 × 10^8^ M and a LOD of 1 × 10^13^ M [82].

Figure 5; Table 1.

#### Colorimetric-based DNAzymes-assisted aptasensors

Colorimetric detection systems are widely used methods for detecting biomolecules and analytes [31, 83]. This detection approach makes use of a color shift that may be observed with the naked eye in the presence of a target without the need for a measuring apparatus [84, 85]. The reported colorimetric-based DNAzymes-assisted aptasensors for cancer biomarker detection are described in the current section and summarized in Table 2.

**Table 2:** Performance of colorimetric-based DNAzyme-assisted aptasensors for cancer detection

Biomarkers/cells	Strategy	Sample	Aptamer Sequence (5′ → 3′)	NM/NPs	LOD	LR	Refs.
PDGF	PGM-based DNAzyme	Saliva	CAGGCTACGGCACGTAGAGCATCACCATGATCCTGTTTT	Fe_3_O_4_ NPs	0.11 fM	3.16 × 10^− 16^ M to 3.16 × 10^− 12^ M	[86]
CEA	Split DNAzyme/Hemin/ABTS	Saliva	GGGTAGGGCGGGTTGGG	-	1 ng mL^− 1^	1–50 ng mL^− 1^	[87]
MUC1	Magnetic NPs/DNAzyme/hemin/H_2_O_2_/ABTS	Serum	TGGGTAGGGCGGGTTGGGAAA	Magnetic NPs	5.08 nM in a PBS 5.60 nM in serum	50-1000 nM	[93]
VEGF	DNAzyme/hemin/H_2_O_2_/ABTS	Serum	CAGACAAGAGTGCAGGGTTT TTTTTTT	-	1.70 pM	24 pM to 11.25 nM	[94]
K-ras gene	DFA-machine/DNAzyme/hemin/H_2_O_2_/ABTS	Cell suspension	Sense primer: CCTGCTGAAAATGACTGAA Anti-sense primer: CATATTCGTCCACAAAATG	-	10 pM	0.01 to 150 nM	[96]
BRCA1 gene	Pb^2+^-DNAzyme/RCA/HP	Buffer	Target DNA: GAACAAAAGGAAGAAAATC Hairpin probe: AGAAAATCATCTCTTCTCC GAGCCGGTCGAAATAGTGGGTGATTTTCTTCCTTTTGTTC	Magnetic beads (MBs)	3.3 fM	-	[97]
PTK7-positive CCRF-CEM cell/	aptamer sgc8c/HRP-DNAzyme/ABTS	suspension	ssDNA-1: ATCTAACTGCTGCGCCGCCGGG AAAATACTGTACGGTTAGACCCAACCC ssDNA-2: TGGGTAGGGCGGGTTGGGTC TAACCGTACAGTA-3’	-	500 cells	-	[99]

##### PDGF

**Fig. 6:**
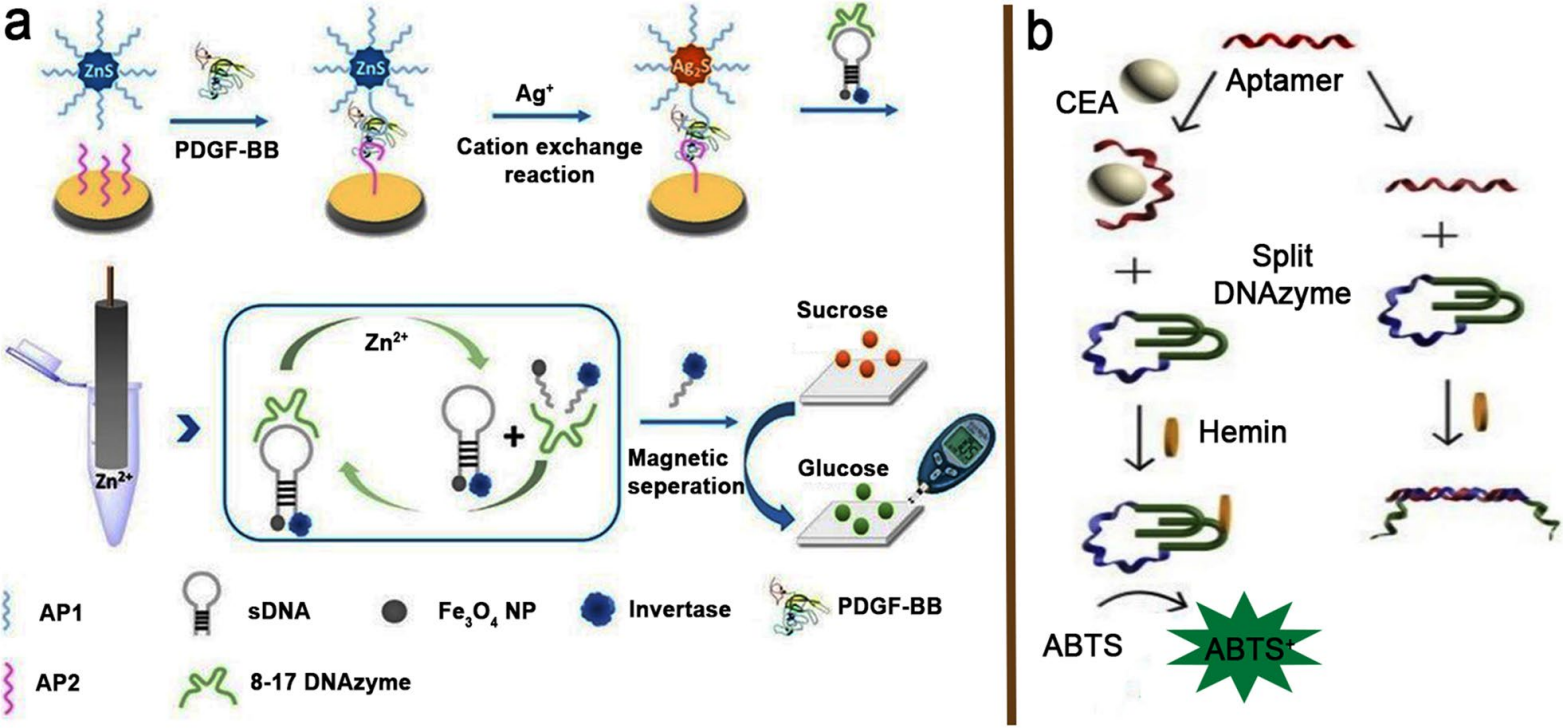
**a** Illustrative process of the colorimetric PGM-based DNAzyme aptasensor for PDGF-BB analyze; **b** Conceptual graphic of a colorimetric DNAzyme aptasensor for CEA detection. Reproduced with permission from [86] and [87]

Hong et al. reported the development of a portable personal glucose meter (PGM) integrating a catalytic and molecular beacon (CAMB) platform with a cation exchange reaction (CX reaction) for the identification of PDGF-BB, sensitively and specifically. Their approach achieved detection amplification in three ways: increased aptamer loading on NPs; release of Zn^2+^ *via* CX reaction; and 8–17 DNAzyme-assisted catalyzing. The binding of PDGF-BB to its aptamer led to the exchange of ZnS to Ag_2_S through a CX reaction, followed by the substrate DNA cleavage in the CAMB platform (Fig. 6a). Sucrose might be converted into glucose using cleaved DNA tagged with invertase, leading to a color change from yellow to green as measured by PGM. The elevated PGM signal correlates with PDGF-BB concentrations ranging from 3.16 × 10^− 16^ to 3.16 × 10^− 12^ M, with a LOD of 0.11 fM [86].

##### CEA

In a study, a colorimetric aptasensor using only two unlabeled oligonucleotides, a CEA aptamer, and a split peroxidase imitating DNAzyme was constructed. The complementary sequence of an aptamer was used to join the halves of the DNAzyme. In the absence of CEA, the conjugation of aptamer and its complementary decreased the DNAzyme catalytic activity. Following aptamer detection of CEA, the oligonucleotide duplex was not generated, inducing the peroxidation and producing the colored ABTS^+^ (Fig. 6b). The aptasensor can detect CEA at LR from 1 to 50 ng mL^− 1^, with a LOD of 1 ng mL^− 1^ [87].

##### Mucin 1 proteins (MUC1)

**Fig. 7:**
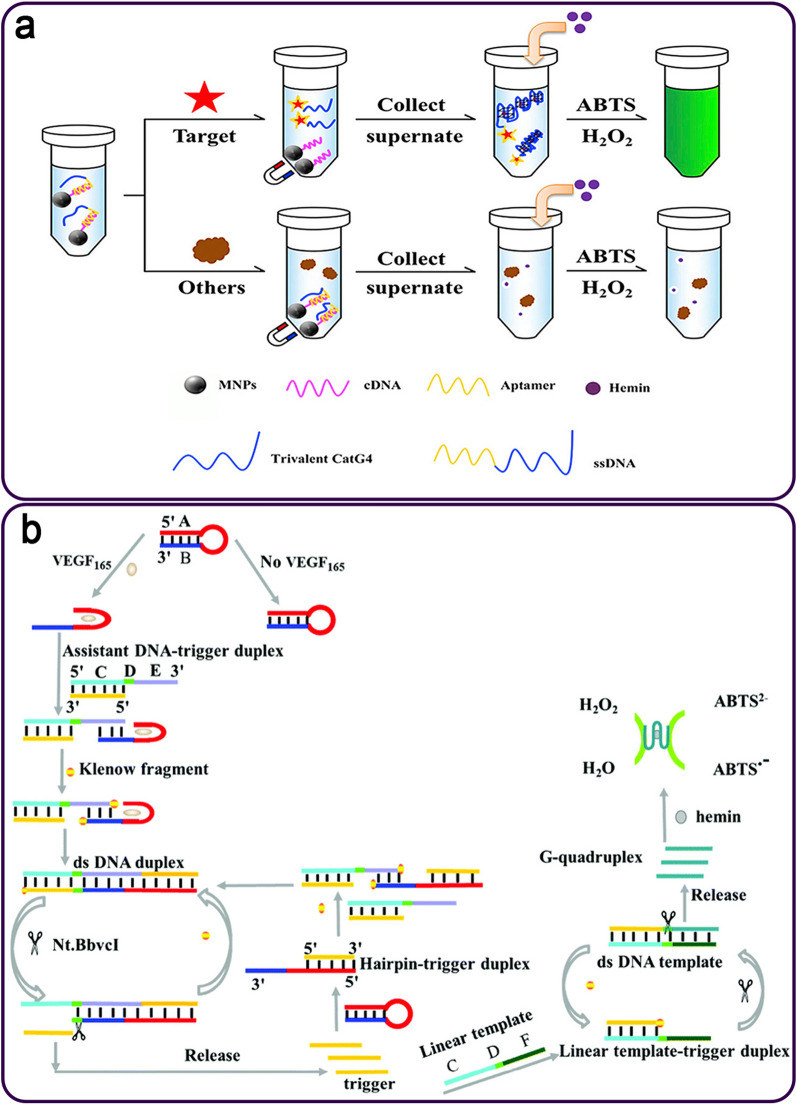
**a** A colorimetric aptasensor for the MUC1 detection using MNPs and DNAzyme catalyzed the H_2_O_2_-related oxidation of the ABTS; **b** The ABTS-based colorimetric DNAzyme-assisted aptasensing of VEGF_165_. Reproduced with permission from [93] and [94]

MUC1 is a glycosylated transmembrane phosphoprotein found on the apical surface of the majority of normal epithelial cells [88, 89]. It is typically expressed in hematopoietic and secretory epithelial cells [90], but is inappropriately overexpressed in breast, colon, prostate, epithelial ovarian, lung, and pancreatic malignancies [38]. These findings point to MUC1 as a promising candidate for cancer diagnosis, treatment, and prognosis [91, 92]. It has been reported as the marker model to show the feasibility of using the DNAzyme-assisted aptasensor. One group presented an aptasensor for colorimetric identification of MUC1 that was made up of magnetic NPs (MNPs), biotin-modified cDNA, and a label-free ssDNA containing an aptamer and a trivalent HRP-like DNAzyme. This platform catalyzed the H_2_O_2_-mediated oxidation reaction of the colorless ABTS to a blue-green compound that is visible to the naked eye (Fig. 7a). The aptasensor detected the target in the range of 50-1000 nM, and the LOD of platform in a buffer solution and a 10% serum medium were 5.08 and 5.60 nM, respectively [93].

##### VEGF

Zhang and colleagues introduced a label-free colorimetric aptasensor containing an aptamer-based hairpin probe, a DNA-trigger duplex, and a linear template for sensitive detection of VEGF_165_. In the presence of VEGF_165_, the hairpin probe was hybridized with the DNA-trigger duplex, leading to the release of triggers. The triggers then generated the G4/hemin DNAzymes, which then catalyzed the transformation of ABTS^2−^ to ABTS^•−^ by H_2_O_2_ to produce a color (Fig. 7b). In the absence of target VEGF_165_, no trigger can initiate the production of G4/hemin DNAzyme. The LOD of the designed aptasensor was 1.70 pM, with a LR from 24.00 pM to 11.25 nM [94].

##### Cancer-related genes

**Fig. 8:**
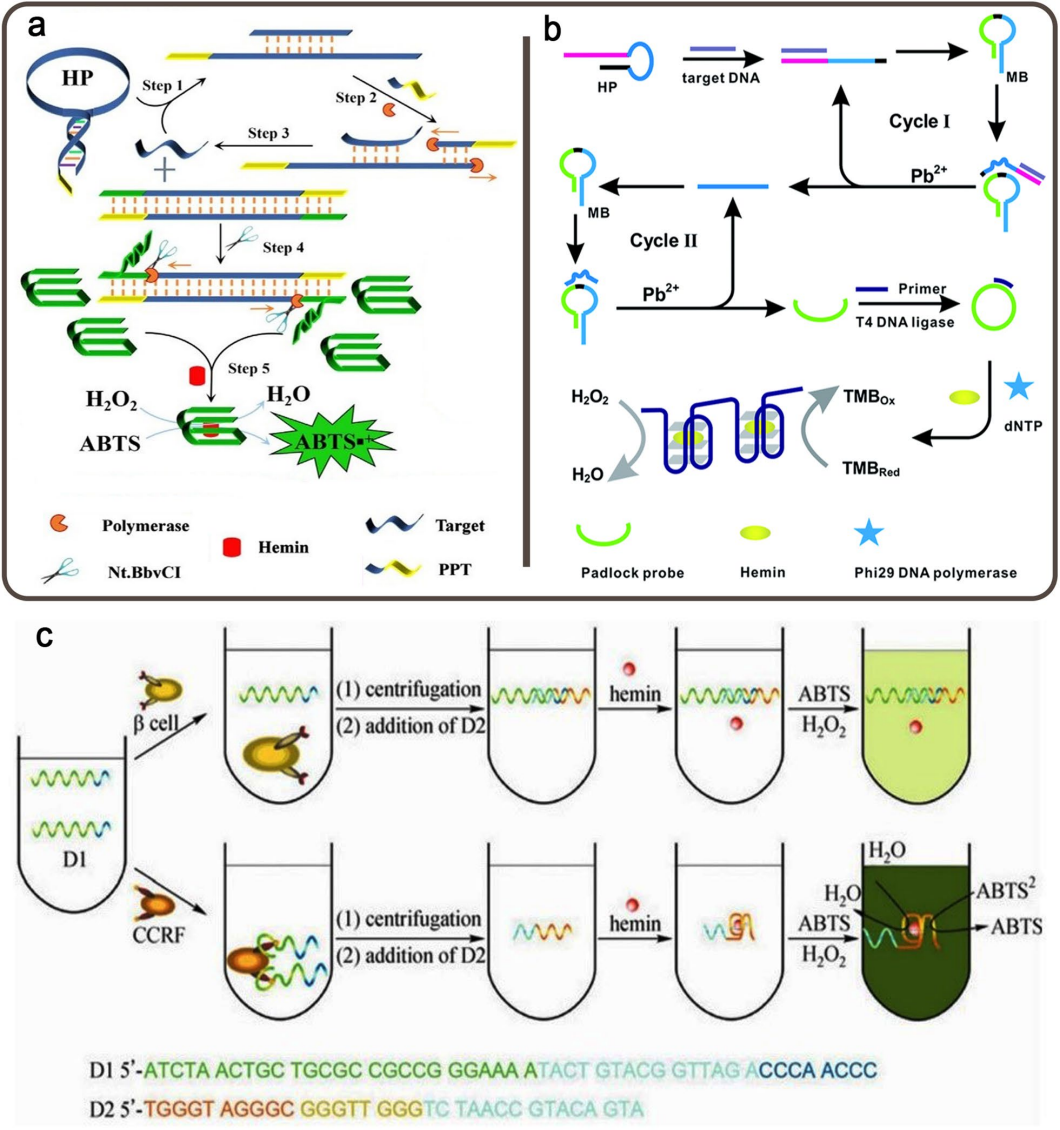
**a** A DNAzyme-assisted colorimetric aptasensing of K-ras gene based on DFA-machine; **b** A DNAzyme and RCA-based colorimetric detection of BRCA1 gene; **c** Schematic representation of the DNAzyme-assisted colorimetric detection of PTK7-positive CCRF-CEM cell. Reproduced with permission from [96], [97] and [99]

The growing interest in RAS genes, particularly K-ras and N-ras, in colorectal cancer (CRC) and non-small-cell lung cancer (NSCLC) patients is related to their potential as predictive biomarkers of response or resistance to targeted therapy. In metastatic CRC patients, K-ras and N-ras mutations, in particular, have a negative predictive role in response to anti-epidermal growth factor receptor (EGFR) monoclonal antibodies [95]. In a study, an aptasensor for the identification of proto-oncogene K-ras based on a DNA molecular machine and dual isothermal circular strand-displacement amplification (D-ICSDA) was designed (Fig. 8a). They created a primer-contained linear polymerization template and a primer-locked hairpin probe (HP) (PPT). HP can hybridize with PPT in the existence of the target gene, making a DNA molecular machine with two functional arms (called the DFA-machine). The polymerase can extend each of the two probes in this machine. Furthermore, the dual isothermal polymerization is converted into D-ICSDA with the help of nicking endonuclease, resulting in the production of an anti-hemin aptamer. The aptamer/hemin duplex, an HRP-assisted DNAzyme, catalyzed the oxidation of colorless ABTS by H_2_O_2_ to a detectable green color. The platform displayed a LOD of 10 pM with a LR of 0.01 to 150 nM [96]. In another work, a colorimetric aptasensing method based on DNAzyme and RCA was developed for early recognition of the BRCA1 gene. The platform benefits from a hairpin probe (HP) that includes the DNA sequence as the identification element and the caged 8–17 DNAzyme part in the loop section as a trigger of the next reaction. Upon adding the target DNA, the DNAzyme was released from the HP, leading to the cleavage of MBs and triggered the RCA reaction in the presence of the Pb^2+^ cofactor. G4/hemin DNAzyme may be formed from the amplified RCA products and used as a direct signal readout element (Fig. 8b). This biosensing method detected BRCA1 down to 3.3 fM with a LR of 7 orders of magnitude [97].

##### Protein tyrosine kinase 7 (PTK7)

PTK7 is a transmembrane receptor highly expressed on human acute lymphoblastic leukemia (CCRF-CEM cells) which can be targeted by aptamer sgc8c [98]. Zhu et al. identified CCRF-CEM cells *via* Sgc8c/DNAzyme. The cancer cell-binding Sgc8c aptamer was used as the identification unit for PTK7-positive target cancer cells, while HRP-like DNAzyme was applied as the probing component to generate ABTS-related colorimetric signals (Fig. 8c). The platform could detect 500 CCRF-CEM cells with high accuracy without the need for expensive equipment or the modification or labeling of DNA sequences [99].

Colorimetric assays are currently being used for cancer biomarkers employing antibody-based assays such as ELISA [100]. Colorimetric DNAzyme-assisted aptasensors are still in their early stages when compared to commercial biomarker detection kits. However, they exhibit at least equivalent sensing characteristics. In comparison with nano-aptasensors, ELISA has a restricted detection limit and sensitivity that considers the potential of nano-aptasensors for point-of-care (POC) clinical diagnostics in the coming years. Furthermore, antibody-assisted assays demonstrate several limitations, including the demand for large sample volumes, specialized apparatus, costly antibodies, extended incubation durations, and intricate methods [101, 102]. Metal NPs, particularly AuNPs, may be suitable nominees for the construction of colorimetric equipment because of their larger extinction coefficient in comparison with organic dyes, which provides excellent sensitivity for colorimetric-based detection kits [83].

### Electrochemical-based DNAzymes-assisted aptasensors

An electrochemical-based aptasensor is a small analytical platform including a coated recognizing element (aptamer) on the surface of a transducer to transform a biological interaction into a measurable signal [103]. Electrochemical aptasensors are particularly appealing as biosensors since they prompt tumor detection biomarkers and are rapid, manageable, exceedingly sensitive, and specific to their targets [104, 105]. In the past few years, numerous biological molecules with varying electrode stability and selectivity have been employed to make biomarker-based electrochemical aptasensors. In electrochemical biomarker-based aptasensors, biomolecules with acceptable precision, tolerable stability, and repeatability should be employed. This section and Table 3 summarize the different electrochemical-based DNAzyme-assisted aptasensors reported for cancer detection.

**Table 3:** Performance of electrochemical-based DNAzyme-assisted aptasensors for cancer detection

Biomarkers/Sample	Strategy	Sample	Aptamer Sequence (5′ → 3′)	NM/NPs	LOD	LR	Refs.
CEA	GQD/AuNPs/DNAzyme	Serum	CEAAptamerI:5′–NH_2_–ATACCAGCTTATTCAATT–3′ CEA Aptamer II: 5′–NH2–CCCATAGGGAAGTG GGGGA–3′	GQD/AuNPs	3.2 fg mL^− 1^	10 fg mL^− 1^ to 200.0 ng mL^− 1^	[106]
Exosomes	label-free DNAzyme/RCA/anti-CD63	Plasma	AACCGCCCAAATCCCTAAGAGTC GGACTGCAACCTATGCTATCGTT GATGTCTGTCC	-	9.54 × 10^2^ mL^− 1^	4.8 × 10^3^ to 4.8 × 10^6^ exosomes mL^− 1^	[107]
MUC1	PCN-224-PtNPs/dual-aptamer/HRP/GQH nanoprobe	Blood	AS1411 or MUC1 aptamer	PtNP	6 cells mL^− 1^	20 to 1 × 10^7^ cells mL^− 1^	[108]
P53	G4-hemin DNAzyme, CS-G, AuNPs, and nicking endonuclease	Buffer	S1: NH_2_-C6-TTTTTTTCT GAC GCT GCT CAC G S2: SH-C6-TTTTTCGTGAGCAGC GTCAG	AuNPs	3.0 × 10^16^ M	1.0 × 10^15^-1.0 × 10^9^ M	[113]
HepG2	G4/hemin/ TLS11a aptamer-AuNPs-HRP nanoprobe	Cell suspension	HS-(CH_2_)6-ACAGCATCCCCATGT GAACAATCGCAT TGTGATTGTTACG GTTTCCGCCTCATGGACGTG CTG	AuNPs	30 cells mL^− 1^	10^2^ to 10^7^ cells mL^− 1^	[115]
	ZnO@Au-Pd-HQ-HRP-G4/hemin/TLS11a aptamer nanoprobe	Blood		ZnO@Au-Pd	10 cells mL^− 1^	10^2^ to 10^7^ cells mL^− 1^	[116]
	Pd-Pt nanocage-HRP-cDNA/hemin/G4 DNAzyme/TLS11a aptamer			Pd-Pt nanocage	5 cells mL^− 1^	10 to 1 × 10^6^ cells mL^− 1^	[117]
	MIL-101@AuNPs, hemin/G4 DNAzyme, TLS11a aptamer and HRP			MIL-101@AuNPs	5 cells mL^− 1^	10^2^ to 10^7^ cells mL^− 1^	[118]
K562 cells	Super-sandwich G4 DNAzyme/cDNA	Cell suspension	ATCCAGAGTGACGCAGCAGATCAG TCTATCTTCTCCTGATGGGTTCCTATTTATAGGTGAAGCTGGACACGGTGG CTTAGT-(CH_2_)6-NH_2_	-	14 cells mL^− 1^	14 to 1.4 × 10^6^ cells mL^− 1^	[119]

#### Electrochemical-based DNAzymes-assisted aptasensing of cancer biomarkers

##### CEA

**Fig. 9:**
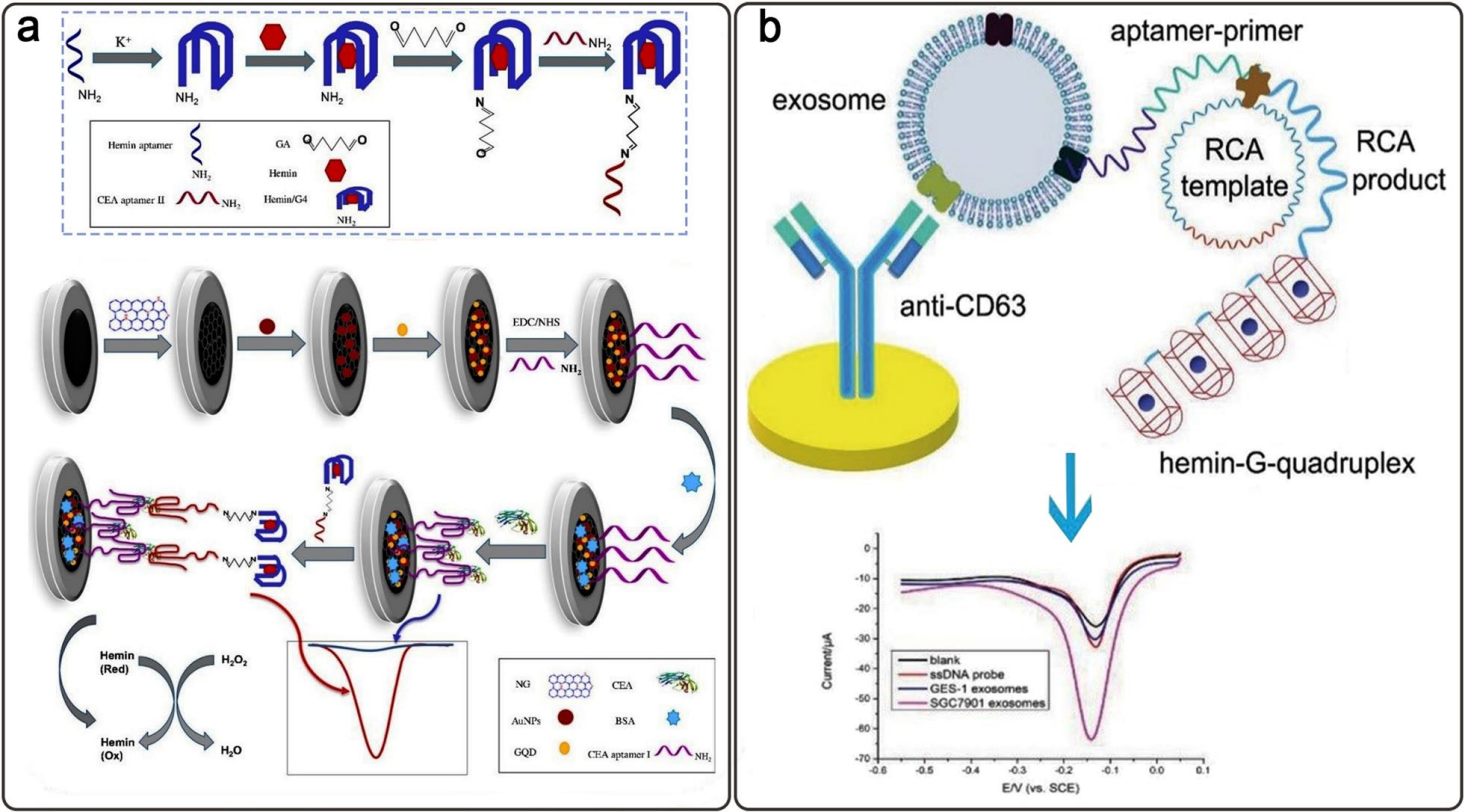
**a** A sandwich-type detection of CEA based on GQD/AuNPs/DNAzyme aptasensor; **b** The scheme of exosomes biomarkers detection based on the label-free DNAzyme-assisted electrochemical aptasensor. Reproduced with permission from [106] and [107]

In an experiment, a sandwich-type electrochemical aptasensor for the sensitive detection of CEA was fabricated. To create an architecture of the GQD/AuNPs/NG/GCE type, first, the surface of a GCE was decorated via nitrogen-doped graphene, and subsequently, AuNPs and GQDs were electrodeposited on it. GCEs are generally used for analytical purposes due to their inertness both chemically and electrochemically and are relatively reproducible. In this platform, the CEA-specific aptamer I was adsorbed on the modified GCE. The CEA-specific aptamer II was linked to hemin-G4 by glutaraldehyde (GA) as a linker and formed the ApII/GA/hemin-G4/DNAzyme. The CEA was sandwiched between them (Fig. 9a). Following that, the hemin-G4 worked as a peroxidase-like DNAzyme, quickly catalyzing the reduction of H_2_O_2_. Differential pulse voltammetry (DPV) was applied to quantify CEA that showed a LR from 10 fg mL^− 1^ to 200 ng mL^− 1^ CEA and a LOD equal to 3.2 fg mL^− 1^ under optimal conditions [106].

An electrochemical label-free DNAzyme aptasensor for the selective identification of stomach cancer exosomal biomarkers was recently presented. This platform included an Au electrode (AuE) with an anti-CD63 antibody modification and a stomach cancer exosome-targeted aptamer. The aptamer was attached to a primer chain that corresponded to a G4 DNA circle (Fig. 9b). The presence of target exosomes caused rolling circle amplification (RCA) and the formation of hemin/G4 HRP-like DNAzyme that catalyzed the reduction of H_2_O_2_ and created electrochemical signals. This aptasensor displayed a LOD of 9.54 × 10^2^ mL^− 1^ and a LR of 4.8 × 10^3^ to 4.8 × 10^6^ exosomes mL^− 1^ [107].

**Fig. 10:**
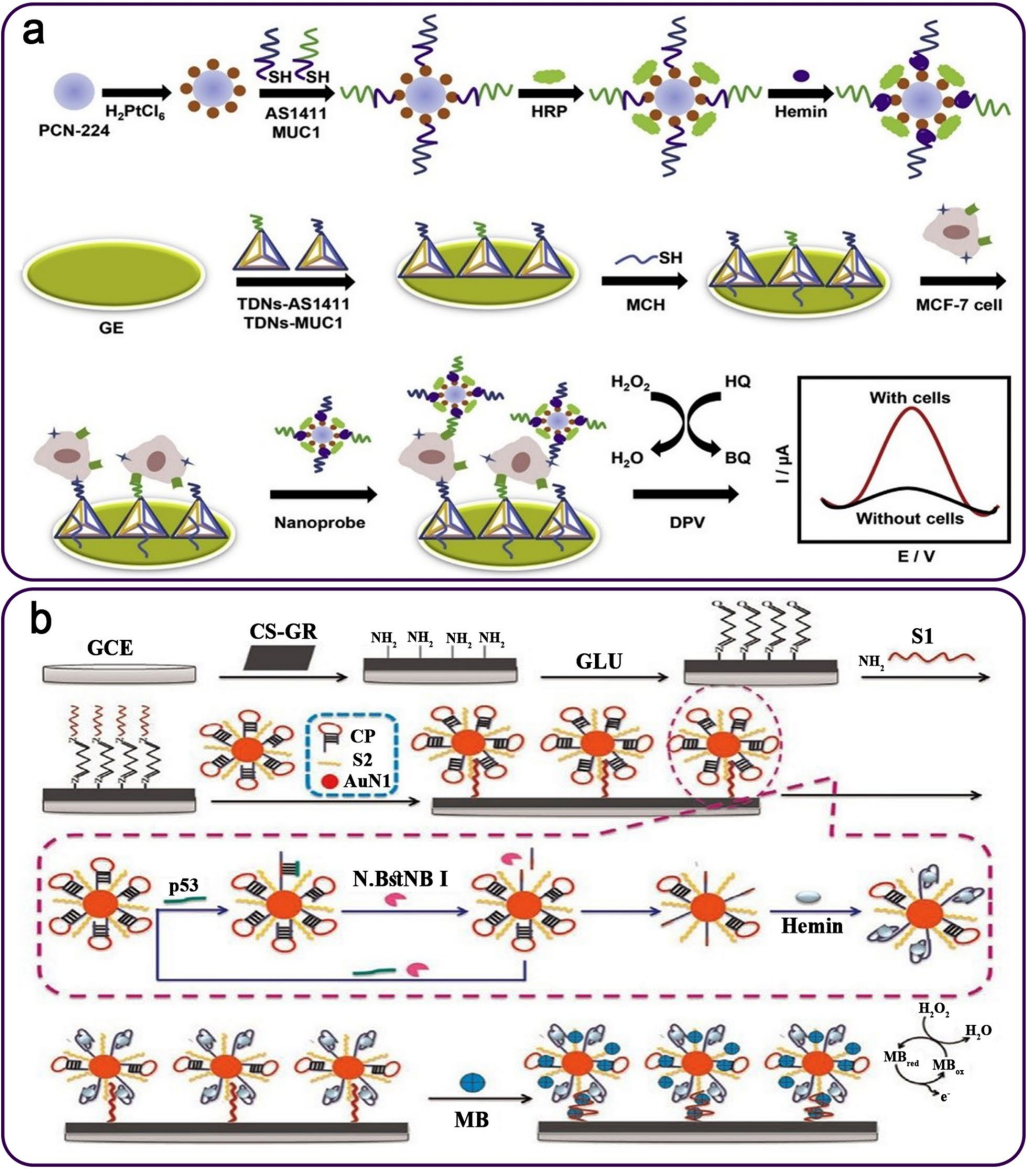
**a** The DNAzyme-assisted sandwich-typed aptasensor for the detection of MCF-7 cancer cell utilizing PCN-224-PtNPs/dual-aptamer/HRP/GQH nanoprobe; **b** A scheme of p53 gene aptasensing assay according to G4-hemin DNAzyme, CS-G, AuNPs, and nicking endonuclease. Reproduced with permission from [108] and [113]

In a study, a sandwich-type cytosensor based on the metal-organic framework (MOF) PCN-224 and tetrahedral DNA nanostructures (TDNs)-linked dual-aptamer (AS1411 and MUC1) was designed to assess cancer cells. To begin with, TDNs-AS1411 and TDNs-MUC1 were immobilized on the AuE surface as bio-recognition components to trap MCF-7 cancer cells. The unique nanoprobes containing PCN-224 coated with PtNPs and modified by the dual aptamer, HRP, and G4/hemin DNAzyme were connected to trapped MCF-7 cells (Fig. 10a). The nanoprobes were used to accelerate the electrochemical signal using the H_2_O_2_-mediated oxidation of hydroquinone (HQ). The findings indicated that the aptasensor’s LR extended from 20 to 1 × 10^7^ cells mL^− 1^, with a LOD of 6 cells mL^− 1^ [108].

P53 is a gene that produces a protein that is present inside the nucleus of cells and regulates cell division and death. Cancer cells may develop and spread in the body because of mutations (changes) in the p53 gene [109, 110]. There have been a substantial number of studies established over the last ten years pertaining to the expression of p53 isoforms in a variety of common malignancies, such as renal cell carcinoma, ovarian cancer, breast cancer, and hepatocellular carcinoma [111]. Overall, p53 isoforms play important roles in carcinogenesis and may serve as biomarkers and therapeutic targets [112]. A label-free G4/hemin DNAzyme aptasensing platform for detecting the p53 gene as a target DNA was introduced based on the chitosan–graphene (CS-G) modified electrode and AuNPs-DNA conjugates (Fig. 10b). The cyclic production of p53 by a nicking endonuclease (N.BstNB I) could be formed into G4-hemin DNAzyme, which may lead to substantial signal amplification in the existence of H_2_O_2_ and the oxidation-reduction reaction of adsorbed MB. With a LR of 1.0 × 10^15^-1.0 × 10^9^ M and a LOD of 3.0 × 10^16^ M, MB’s DPV signals quantified the p53 concentrations [113].

#### Electrochemical-based DNAzymes-assisted aptasensors for cancer cell detection

In some research, electrochemical-based DNAzyme aptasensing platforms have been used for the detection of cancer cells using aptamers that are capable of specific binding to the unknown membrane proteins of tumor cells.

The TLS11a liver cancer-specific aptamer has been used as a targeting agent in some electrochemical-based DNAzyme-assisted aptasensors. It has been demonstrated that the target molecule of TLS11a is a membrane protein that can be overexpressed in liver cancer cells [114]. Regarding the specific binding of TLS11a aptamer to liver cancer cells, some researchers have been interested in using electrochemical-based DNAzyme aptasensing platforms for HepG2 (human liver hepatocellular carcinoma cells) cancer cell detection (Table 3).

**Fig. 11:**
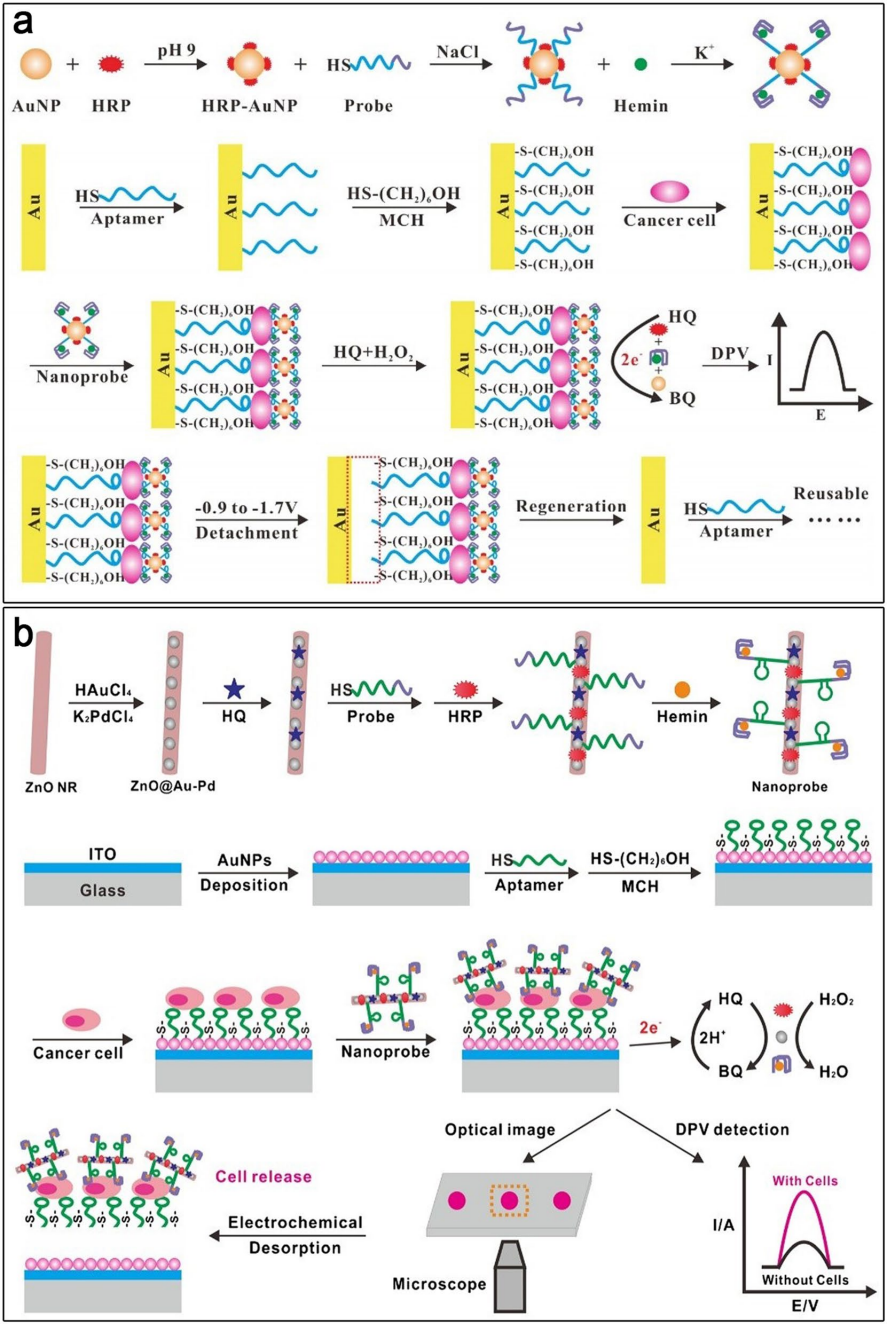
**a** An electrochemical detection of HepG2 cells using G4/hemin/aptamer-AuNPs-HRP nanoprobe; **b** The processes of HepG2 cells aptasensing detection based on ZnO@Au-Pd-HQ-HRP-G4/hemin/aptamer nanoprobe. Reproduced with permission from [115] and [116]

An aptazymes-based electrochemical cytosensor was designed according to a dual recognition and signal amplification technique for the sensitive detection of HepG2 cells. First, a thiolated TLS11a aptamer covalently linked to an AuE. Meanwhile, a G4/hemin/aptamer and HRP-modified AuNPs (G4/hemin/aptamer-AuNPs-HRP) were applied as nanoprobe (Fig. 11a). The HepG2 cancer cells were trapped using nanoprobes as recognizing probes to create an aptamer-cell-nanoprobe sandwich-like nanosystem on an AuE surface. The suggested electrochemical aptasensor displayed an LR of 1 × 10^2^ to 1 × 10^7^ cells mL^− 1^ as well as a LOD of 30 cells mL^− 1^ via the electrochemical impedance spectroscopy (EIS) and cyclic voltammetry (CV) methods [115]. In this regard, a DNAzyme-related technique for identifying and quantifying HepG2 cells in the blood has been reported. In this chip-based electrochemical aptasensor, HepG2 cells were sandwiched between the hybrid nanoprobes, and a cell-targeting TLS11a aptamer was immobilized on an indium tin oxide (ITO) electrode/AuNPs. The hybrid nanoprobe comprised a HQ as an electrochemical probe, HRP, and an aptamer/hemin/G4 complex adsorbed on Au/palladium-functionalized ZnO nanorods (ZnO@Au-Pd) (Fig. 11b). The nanoprobes could magnify the voltammetric signal and capture the target cells. The electrode had a LR of 10^2^ to 10^7^ HepG2 cells mL^− 1^ and a LOD of 10 cells mL^− 1^ [116].

**Fig. 12:**
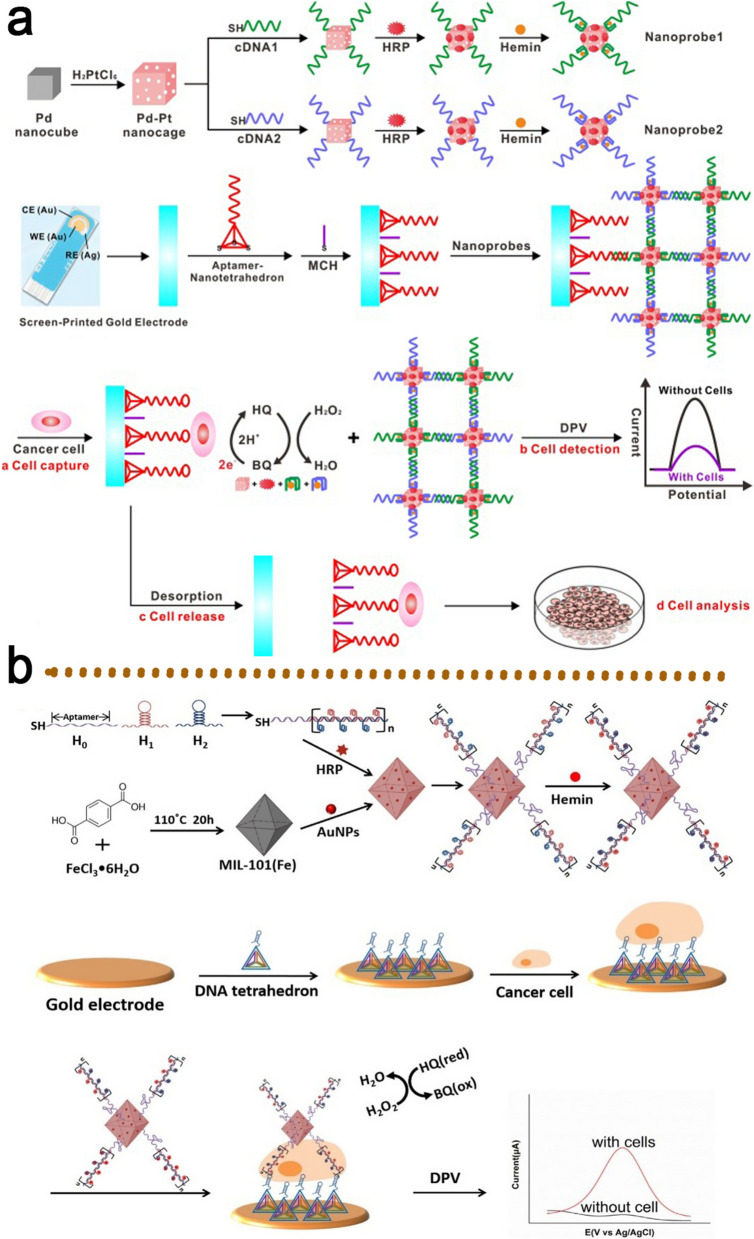
**a** Schemes illustration of CTCs of HepG2 detection by Pd-Pt nanocage-HRP-cDNA/TLS11a aptamer/hemin/G4 hybrid nanoprobes; **b** An aptasensor based on DNA tetrahedron-cell-nanoprobe (MIL-101@AuNPs, hemin/G4 DNAzyme, and HRP) sandwich-like for the detection of HepG2 cell. Reproduced with permission from [117] and [118]

In another work, a label-free and competitive DPV-based aptasensor for the effective identification of circulating tumor cells (CTCs) was generated from HepG2 cells. CTCs, as a kind of tumor-derived cell, are released into the blood circulation. The detection of CTCs has been considered as an attractive diagnostic approach for cancer patients [35]. To begin with, the TLS11a aptamer was mounted on a screen-printed AuE (SP-AuE) surface to capture the HepG2 cells. Then, using DNA hybridization, hybrid nanoprobes of Pd-Pt nanocages functionalized with cDNA, hemin/G4 DNAzyme, and HRP were linked to the SP-AuE-loaded aptamer, which resulted in the establishment of dendritic structure (DS) nanoprobes (Fig. 12a). In the existence of HepG2 cells, they can interact with aptamer probes, causing the detachment of DS nanoprobes from the SP-AuE. With a LOD of 5 cells mL^− 1^, this approach has ultrahigh selectivity and sensitivity regarding the identification of HepG2 cells [117]. In 2018, Chen et al. introduced a DNAzyme nano-aptasensor for the detection of HepG2 cells using a multibranched HCR amplification approach. In their platform, the immobilizing DNA tetrahedron on the AuE can be specifically captured by HepG2 cells (Fig. 12b). Then, the functional hybrid nanoprobes consisting of MIL-101@AuNPs, hemin/G4 DNAzyme, and HRP were attached to the captured-HepG2 cell and built a DNA tetrahedron-HepG2-nanoprobe sandwich-like structure on the AuE surface. These hybrid nanoprobes performed selective discrimination and enzymatic signal amplification at the same time. This cytosensor has a LOD of 5 cells mL^− 1^ with a LR of 10^2^ to 10^7^ cells mL^− 1^ [118].

In a study for cancer cell detection based on aptamer-based competition and a super-sandwich G4 DNAzyme amplification technique, Lu et al. created a new aptasensor for electrochemical detection of K562 cancer cells. Because the aptamer has a higher affinity for cancer cells compared to its corresponding oligonucleotide, the complementary oligonucleotide may be easily substituted. Consequently, their method detected cancer cells indirectly by measuring the amount of DNA released, which is equal to the quantity of K562 cells. Sensitivity was greatly increased using the super sandwich G4 DNAzyme amplification technique, with LOD as low as 14 cells [119].

## Challenges of DNAzyme-assisted aptasensing of cancer biomarkers

It has been demonstrated that cancer biomarkers may be discovered in small amounts in the circulation before diagnosing cancer, but their levels increase in parallel with tumor growth. The identification of low-level cancer biomarkers in early-stage cancer is critical for diagnosis, screening, and prognosis, as well as predicting therapy outcomes. Recent approaches, in contrast to older methods, may detect cancer biomarkers at nanomolar and subnanomolar concentrations without needing a pre-concentration step. On a more general basis, cancer biomarkers can be found in a variety of isoforms: For example, MUC1 present in tumors (tMUC1 or TA-MUC1) has a different structure than normal MUC1 [103]. The immune system recognizes altered glycosylated epitopes in tMUC1, while broad branches of glycosylation prevent antigenic detection in normal MUC1. In other cases, VEGF contains four distinct isoforms, including VEGF_121_, VEGF_189_, VEGF_165_, and VEGF_206_, which are produced by alternate exon splicing of the VEGFA gene [120]. These characteristics were taken into account while designing aptamers and aptasensors for differentiating normal from malignant cells as well as cancer biomarker isoforms.

Recently, a high degree of research has been focused on the field of DNAzyme-based detection. Compared to common detection methods, DNAzyme-based biosensors have shown high detection sensitivity, greater stability, and strong accuracy at a lower cost that can enhance the sensing cascades. In many DNAzyme-assisted aptasensors, the immobilization of DNAzymes onto an underlying substrate can improve detection sensitivity through the decreeing of background noise and has resulted in the development of reusable sensors. To optimize the performance of DNAzyme-assisted aptasensors, the nature of the underlying substrate, the choice of an appropriate immobilization strategy, and the signal transduction method must all be considered. A wide range of nanomaterials and NPs possess distinct properties that make DNAzyme-assisted aptasensors suitable for particular sensing systems [19]. In addition, the alteration of DNAzyme activity concerning fluorophore photobleaching and temperature are drawbacks that need to be addressed to ensure valid detection. Some chemical processes can be catalyzed by DNAzymes. HRP-mimicking DNAzyme is commonly used in optical and electrochemical aptasensing platforms. It has a unique G-4 structure with an added hemin that may oxidize the ABTS^2−^ to the colored ABTS^−^ or create CL by oxidizing the luminal with H_2_O_2_, while also exhibiting high catalytic activity toward H_2_O_2_.

The majority of research that employed DNAzyme-assisted aptasensors to identify cancer biomarkers was conducted in vitro. Therefore, the DNAzyme-assisted aptasensors to be able to detect cancer biomarkers in vivo and in complicated clinical specimens are still at the beginning and have a long way to go. As far as we can tell, no report on DNAzyme aptamer approaches that could be used in the clinic has been published. Because most human cancers include many biomarkers, the interference of developed DNAzyme aptasensors with diverse biomarkers existing in cancer cells continues to be a challenge to overcome for specific biomarker detection. Furthermore, determining the local quantities of signals released by cancer cells in vivo is challenging. The simultaneous detection of various biomarkers in actual samples might be addressed by optimizing operating variables such as temperature, pH, ionic strength, and buffer components, DNAzyme function, as well as the modification or functionalizing the sensor surface by several aptamers. For both in vivo and complicated clinical sample analysis, the creation of various detection systems combined with NPs should be addressed.

As mentioned in this review, NPs-based DNAzyme-assisted aptasensors (DNAzyme-assisted nano-aptasensors) have been widely used to build a variety of platforms for cancer biomarker diagnosis. For a variety of single or complex targets, the sensitivity, selectivity, and speed of the existing aptasensors may be enhanced by the conjugation of NPs with highly selective aptamers. In this study, nano-aptasensors were demonstrated to have a lower detection limit than aptasensors, which was described in this study. Among NPs, AuNPs can generally be used in the fabrication of DNAzyme-assisted aptasensors, especially in colorimetric platforms, for the detection of cancer biomarkers due to the fact that AuNPs raise the sensitivity and enhance the output signal of sensors [19, 39]. GO can be added to fluorescence aptasensors to improve sensitivity and lower background signals because of its super-quenching abilities [121]. As such, QDs are also being taken into consideration for use in optical DNAzyme aptasensing strategies owing to their adaptability concerning bioactivity, sizes, charges, stability, surface characteristics, and core/shell structure [122]. NPs can boost the output signals of electrochemical aptasensors by altering the surface of electrodes and accelerating electron transfer across the electrode [123]. Therefore, for the precise detection of cancer biomarkers, DNAzyme-assisted nano-aptasensors with excellent sensitivity and specificity can be an ideal option that allows binding to different sites of the same target via a pair of receptors.

As mentioned in this review, several optical and electrochemical-based DNAzyme-assisted aptasensors can be useful for cancer biomarkers and cancer cells in a variety of ways. The following description compares the benefits and drawbacks of these aptasensing methods. Optical methods have many advantages, including real-time, quantitative, direct, and label-free detection. Colorimetric-based platforms have potential applications in portable systems due to simple fabrication without the need for advanced instruments and their results can be observed directly by the naked eye. However, optical-based detection suffers from limitations, including the fact that the luminescence and fluorescence-based platforms are frequently expensive and their fabrication is time-consuming and sophisticated [124]. Electrochemical biosensors have a number of benefits, including being quantitative, reusable, and requiring only a small volume of sample or reagent. The limitations of control over the working electrode’s surface at higher currents and the possibility of falsely positive results originating from electrolytes are some of the drawbacks of electrochemical biosensors [125, 126].

## Conclusions and future perspectives

Aptasensors have recently gotten a lot of attention for their accuracy and speed in detecting critical cancer indicators. Aptamers’ unique properties, like simple synthesis, greater sensitivity, specificity, and high stability, make them appealing candidates for constructing a variety of sensing tools. They are attractive candidates for sensing because of their reproducibility and scalability via solid-phase synthesis (without batch variations), reprogrammability by directed evolution and local sequence modifications, and their ability to achieve high catalytic turnover independent of any auxiliary proteins. Another fascinating advantage of employing aptasensors is the prospect of building aptasensor arrays, which would enable the simultaneous detection of a large number of cancer biomarkers. In this regard, enhancing the aptamer’s precise binding to a particular target aids in resolving this issue. The rational design of DNAzymes and the selection of their targets can be streamlined with the help of rule-based algorithms, which can also be integrated with RNA 3D modeling and high throughput screening.

Despite several efforts to establish the usefulness of cancer biomarker aptamers in various sensing techniques, more work remains to be done to offer DNAzyme aptasensors for cancer biomarkers as a viable strategy for point-of-care diagnostics. For example, one of the aptamers identified against cancer biomarkers has the potential to recognize cancer biomarkers in animal models, increasing the likelihood of approval through in vivo investigations and subsequent entry into the preclinical trial phase. The nuclease breakdown, toxicity, and restricted metabolism of aptamers in a physiological milieu are highlighted as major drawbacks that prevent aptamer DNAzyme conjugates from being used more widely as treatments.

The incorporation of NPs with DNAzyme aptasensing technologies has resulted in significant increases in aptasensor performance due to their unique functional capabilities. This characteristic can be used to create ultra-sensitive and multi-parametric DNAzyme-assisted aptasensors. In this line, nano-aptasensors have achieved detection limits spanning from picomolar to attomolar (10^− 18^ moles per liter) levels, promising a new age of in vitro and in vivo cancer detection. However, for in vivo detection of cancer biomarkers, a general issue about the bio-safety of NPs should be considered. Given the versatile nature of hemin/G4 DNAzyme sensing platforms, significant progress in the development of them is expected to occur in tandem with on-chip and microfluidic-based sensing structures with possibly important clinical diagnostic claims.

## Data Availability

Not applicable.

## Abbreviations

AuNPs: Gold Nanoparticles
AuE: Au electrode
AuNRs: Gold nanorods
CAMB: Catalytic and molecular beacon
CEA: Carcinoembryonic antigen
CL: Chemiluminescence
CRA: Cascade recycling amplification
cDNA: Complementary DNA
CV: Cyclic voltammetry
CTCs: Circulating tumor cells.
CRET: CL resonance energy transfer
CCRF-CEM: Human acute lymphoblastic leukemia
CHA: Catalytic hairpin assembly
DS: Dendritic structure
DPV: Differential pulse voltammetry
ECL: Electrochemiluminescence
EIS: Electrochemical impedance spectroscopy
ExoIII: Exonuclease III
GQDs: Graphene quantum dot
GCE: Glassy carbon electrode
g-C_3_N_4_ NS: Graphitic carbon nitride nanosheets
G4: G-quadruplex
HQ: Hydroquinone
HCR: Hybridization chain reaction
HepG2: Human liver hepatocellular carcinoma cells
ITO: Indium tin oxide
GA: Glutaraldehyde
LOD: Limit of detection
LR: Linear range.
MB: Methylene blue
miRNAs: MicroRNAs
MPA: Mercaptopropionic acid
MNPs: Magnetic NPs
MOF: Metal–organic framework
NCs: Nanocrystals
ncRNAs: Non-coding RNAs
NPs: Nanoparticles
ODI: Oxalyldiimidazole
PDGF: Platelet-derived growth factor
PEC: Photo ECL
PET: Photoinduced electron transfer
PGM: Portable personal glucose meter
POC: Point-of-care
RCA: Rolling circle amplification
Sa-RECL: Signal-amplified ratiometric ECL
TDNs: Tetrahedral DNA nanostructures
VEGF: Vascular endothelial growth factor
